# Template‐Encoded Assembly and Adaptive Function in Selenium‐Based Molybdenum Chalcoxide Rings

**DOI:** 10.1002/anie.5129627

**Published:** 2026-09-29

**Authors:** Giulia Pellegrino, Alexander Elliott, Yu‐Fei Song, Nuno A. G. Bandeira, Haralampos N. Miras

**Affiliations:** ^1^ School of Chemistry The University of Glasgow Glasgow UK; ^2^ State Key Laboratory of Chemical Resource Engineering Beijing Advanced Innovation Centre for Soft Matter Science and Engineering Beijing University of Chemical Technology Beijing China; ^3^ Departamento de Química e Bioquímica Faculdade de Ciências Biosystems and Integrative Sciences Institute (BioISI) Universidade de Lisboa Lisboa Portugal

**Keywords:** molecular chalcoxides, polyoxometalates, self‐assembly, selenium, host–guest systems

## Abstract

Controlling both structure and function in inorganic self‐assembly remains a major challenge. Here we establish a programmable strategy for constructing selenium‐based molybdenum chalcoxides using the [Mo_2_O_2_Se_2_]^2+^ dimer as a universal building block. The template‐directed assembly process produces a complete family of ring architectures (*n* = 8–16), with nuclearity encoded by the template's geometry and acidity. Selenium incorporation introduces electronic softness that imparts unusual structural flexibility and enables adaptive behaviour. NMR titration experiments reveal host–guest exchange in solution, demonstrating dynamic recognition in these purely inorganic rings. This family of clusters also displays tuneable electrocatalytic hydrogen evolution activity (HER), following the trend Mo_8_ > Mo_12_ > Mo_10_ and consistently outperforming their sulphur analogues. Together, these results define a design framework in which modular assembly, dynamic function, and catalytic activity arise from a common chalcogen‐dependent structural logic, opening new avenues for responsive inorganic molecular materials.

## Graphical abstract

External templates write structural information into Se‐containing molybdenum rings, while selenium's softness confers adaptive host–guest behaviour and catalytic responsiveness, an integrated framework for programmable inorganic function.

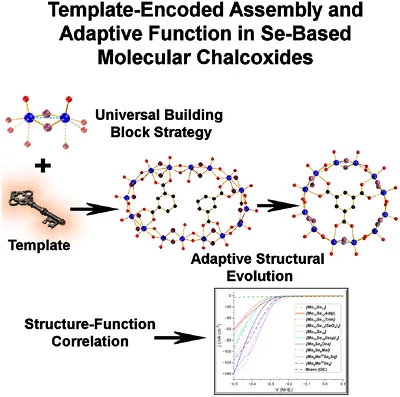

## 1 Introduction

The design of molecular frameworks whose composition and topology can be precisely controlled by programmable self‐assembly is a long‐standing objective in supramolecular and inorganic chemistry [1, 2, 3]. Although polyoxometalates (POMs) and transition‐metal chalcogenides offer exceptional structural and electronic diversity, the rules governing their nucleation, growth, and functional expression are often empirical. In particular, the development of programmable inorganic systems, where structure, reactivity, and dynamics can be deliberately encoded through external inputs, has lagged far behind that of organic host–guest and metal‐organic systems [4, 5, 6, 7]. Molecular molybdenum chalcogenides represent a compelling yet underexplored platform for such endeavours. Their modular metal–oxo–chalcogen building blocks enable rich redox chemistry, variable coordination geometries, and extended electronic delocalisation [8, 9, 10, 11, 12, 13, 14, 15, 16]. Whereas the sulphur‐based Mo‐S family has been widely investigated [17, 18, 19, 20, 21, 22, 23, 24, 25], selenium analogues remain scarce despite their fundamentally different bonding characteristics. Selenium introduces greater size, polarizability and electronic softness, properties predicted to afford enhanced structural flexibility and dynamic behaviour [26, 27, 28, 29, 30, 31, 32, 33]. Yet the absence of general synthetic strategies and predictable mechanistic principles has left Se‐containing clusters effectively inaccessible as programmable materials (Figure 1).

**FIGURE 1:**
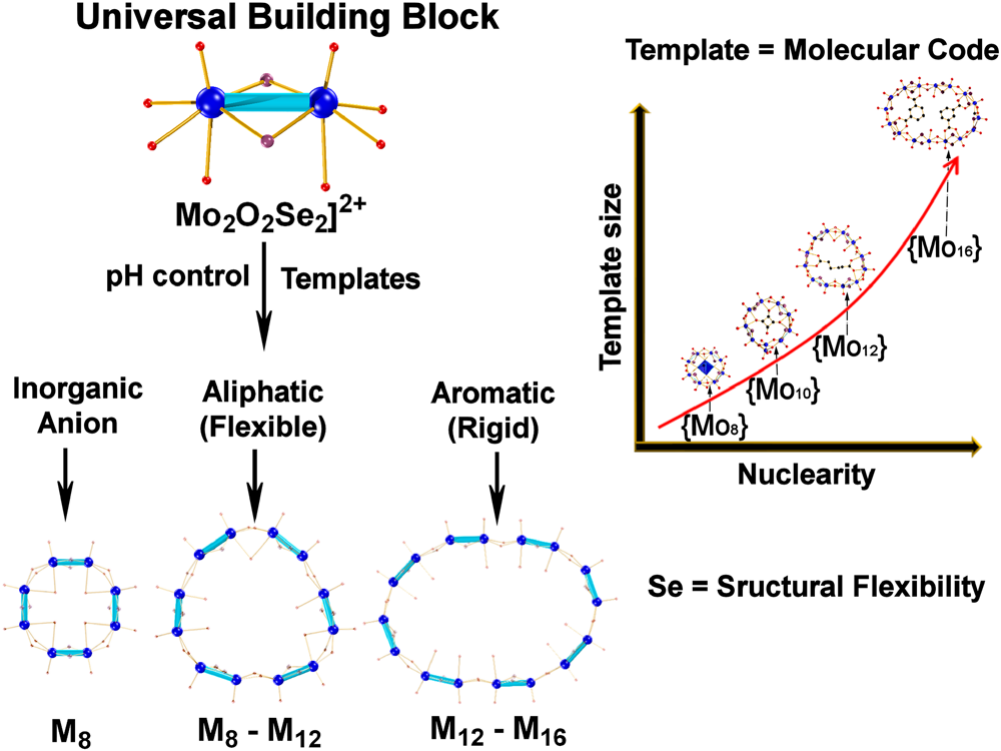
Conceptual illustration of programmable self‐assembly of Se‐based molybdenum chalcoxides. The [Mo_2_O_2_Se_2_]^2+^ dimer acts as a universal module directing the formation of ring architectures with nuclearities ranging from {Mo_8_Se_8_} to {Mo_16_Se_16_} depending on the template's size and geometry. Inset: correlation between template and cluster nuclearity.

The [Mo_2_O_2_Se_2_]^2+^ unit functions as a modular building block whose condensation can be steered by template geometry and acidity, enabling predictable formation of a family of selenium‐based chalcoxide rings spanning nuclearities from {Mo_8_} to {Mo_16_}. The increased electronic softness of selenium further enables adaptive structural behaviour, including template‐triggered structural evolution between distinct ring architectures. These assemblies also display nuclearity‐dependent electrocatalytic hydrogen evolution activity, revealing a direct structure‐function relationship across the ring family. Together, these results establish a unified framework linking programmed self‐assembly, adaptive host–guest chemistry, and catalytic function in molecular chalcogenide clusters. Establishing such predictable relationships between template properties, cluster nuclearity, and emergent function remains a central challenge in the design of adaptive inorganic assemblies.

## 2 Results and Discussion

### 2.1 Modular Self‐Assembly and Template Control

To understand how template geometry and chemical environment govern ring nuclearity and function, we first establish the [Mo_2_O_2_Se_2_]^2+^ building block and then examine how different templates direct the formation, dynamics, and catalytic behaviour of the resulting macrocycles. The series of Se‐based chalcoxides reported here demonstrate that the [Mo_2_O_2_Se_2_]^2+^ dimer operates as a universal structural module capable of assembling into molecular rings of tuneable nuclearity and topology. Across all systems, the fundamental aggregation motif involves edge‐sharing of dimeric units mediated by chalcogen bridges, a process analogous to polymerisation at the molecular level. The resulting architectures, ranging from compact {Mo_8_Se_8_} species to expanded {Mo_16_Se_16_} macrocycles, reveal a continuous structural progression that can be rationalized in terms of template geometry, charge density, and hydrogen‐bonding complementarity. The fundamental dimeric building block, [Mo_2_O_2_Se_2_]^2+^, is generated by oxidative activation of a diselenide precursor followed by condensation under mild basic conditions [34, 35] (see SI, Section 8). The persistence of this building block should nevertheless be considered within the pH‐dependent speciation of the reaction medium. Protonation and deprotonation of the selenium‐containing species, together with hydrolysis and condensation equilibria, influence the concentration and reactivity of the building unit and ultimately determine which higher‐nuclearity architectures become accessible. Thus, [Mo_2_O_2_Se_2_]^2+^ is best regarded as the fundamental molecular synthon under the assembly conditions rather than as a pH‐independent species. Changes in pH consequently alter not only the rate of condensation but also the distribution of accessible cluster nuclearities.

The self‐assembly is driven by a condensation process induced by a basic medium. The self‐assembly process in the absence of template moieties, leads to the formation of two distinct rings: {Mo_10_}[(Mo_2_O_2_Se_2_(OH)_2_(H_2_O))_5_I_2_](TMA)_2_ and {Mo_12_}, [(Mo_2_O_2_Se_2_(OH)_2_(H_2_O))_6_], (Figure 2). The {Mo_10_Se_10_} is synthesised by the direct addition of lithium hydroxide to an acidic solution of [Mo_2_O_2_(*μ*‐Se)_2_]^2+^ within the pH range of 1.5–2 and ∼5 for the {Mo_10_} and {Mo_12_} species respectively. It is noteworthy that the formation of the {Mo_10_} structure forms under increased ionic strength conditions by the addition of ammonium chloride. In the absence of structure directing agents, the {Mo_10_} and {Mo_12_} species seem to be the thermodynamic minima of the assembly reaction coordinates. However, the structure and nuclearity can be precisely directed by a template‐driven assembly process [36]. Having established the [Mo_2_O_2_Se_2_]^2+^ dimer as a robust modular unit, we next examined how external molecular inputs influence its aggregation. Due to the fact that the Se‐based framework tolerates multiple coordination geometries, we anticipated that a template identity would play a decisive role in dictating nuclearity. This prompted a systematic study of how geometric, electronic, and acidic features of the template determine the direction of the self‐assembly pathway [36].

**FIGURE 2:**
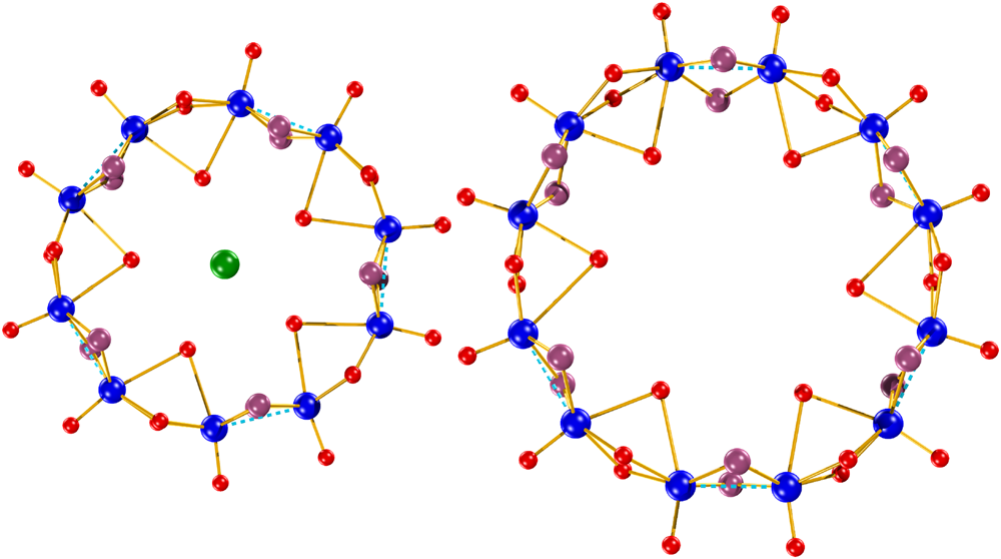
Ball‐and‐Stick representation of: {Mo_10_} (LEFT) and {Mo_12_} (RIGHT). Colour code: Mo, blue; Se, purple; I, green; O, red; Mo–Mo bonds, dashed light blue lines.

A comprehensive assortment of templates is available, featuring dicarboxylates [37, 38, 39, 40, 41, 42, 43, 44, 45, 46] and inorganic anions [47, 48], which facilitate the formation of numerous structures. Although most templates result in mono‐templated or bi‐templated flat ring‐shaped structures, the strategic selection of a larger template can enable the design of 3D structures [49, 50] and, in certain instances, chiral configurations [51, 52]. The strength of the template's interaction strongly dictates the structural features, the nuclearity, and the reorganisation of the dimeric units and their oligomeric intermediates to accommodate the template. In general, smaller templates induce a contraction of the ring and provide access to smaller nuclearities, whereas larger templates lead to an expanded structural motif with higher nuclearities. Finally, linear templates induce distortion of the ring‐shaped structure into elliptical morphologies [38, 39, 40, 41, 42, 43, 44].

#### Inorganic Template Effect

Inorganic templated structures appear to be the products of kinetically favourable self‐assembly processes. Occasionally, these templates can also be generated in situ through oxidative processes influenced by the presence of atmospheric oxygen during crystallisation. For example, the ring shaped moieties [(Mo_2_O_2_Se_2_(OH)_2_)_4_Mo^VI^O_4_(H_2_O)_2_](TMA)_2_, {Mo_8_Mo^VI^}, and [(Mo_2_O_2_Se_2_(OH)_2_)_6_(SeO_4_)_2_(H_2_O)_2_]K_2_, {Mo_12_(SeO_4_)_2_}, can be synthesised through the intentional addition of Na_2_MoO_4_ and SeO_4_^2–^ respectively, but are also found when solutions are allowed to age in the absence of these templates (Figures S1 and S2).

#### Aliphatic Template Effect

This type of organic templates offers considerable benefits in terms of flexibility and conformational adaptability, attributed to their long C–C chains. In this case we introduce three different dicarboxylic acids: oxalic acid (*C*_n_ = 2), malonic acid (*C*_n_ = 3), and adipic acid (*C*_n_ = 6) into solutions of the dimeric building block. The addition of LiOH triggers the condensation process which facilitates the deprotonation of the aliphatic organic templates. This initiates a template driven assembly process and modulates the size and nuclearity of the isolated molecular rings. More specifically three rings were isolated, namely: {[Mo_2_O_2_Se_2_(OH)_2_]_4_(C_2_O_4_)}(NMe_4_)_2_, {Mo_8_Ox}, {[Mo_2_O_2_Se_2_(OH)_2_]_4_C_3_H_2_O_4_}(NMe_4_)_2_, {Mo_8_Mal} and {[Mo_2_O_2_Se_2_ (OH)_2_]_6_C_6_H_8_O_4_}(NMe_4_)_2_, {Mo_12_Adip} at a pH value of 5 (Figures S3‐S5). While simple inorganic anions and aliphatic carboxylates revealed fundamental correlation in ring formation, they provided only a limited geometric range. To assess whether more complex spatial constraints could further steer nuclearity, we employed rigid aromatic templates.

#### Aromatic Template Effect

To probe whether nuclearity can be encoded not only by template size but also by template rigidity and directional coordination, we next investigated aromatic carboxylates as structure‐directing templates. In contrast to flexible aliphatic dicarboxylates, aromatic ligands impose fixed coordination vectors and reduced conformational freedom, thereby providing a stringent test of programmability in the Se‐rich assembly. Under these conditions, the [Mo_2_O_2_Se_2_]^2+^ building block consistently assembles into macrocycles whose nuclearity and curvature reflect the geometric constraints of the guest.

Following a similar synthetic approach we prepared and characterised three additional molecular selenium based chalcoxides using aromatic ligands as templates, namely: squaric, isophthalic and trimesic acids with formulae [(Mo_2_O_2_Se_2_)_4_(OH)_6_(C_4_O_4_)(Mo^VI^O_4_H)_2_](NMe_4_)_2_, {Mo_8_(Mo^VI^)_2_Sq}, [(Mo_2_O_2_Se_2_(OH)_2_)_8_(C_8_H_4_O_4_)_2_(H_2_O)_2_](NMe_4_)_4_, {Mo_16_(Isop)_2_} and, [(Mo_2_O_2_Se_2_(OH)_2_)_6_(C_9_O_6_H_4_)](NMe_4_)_2_, {Mo_12_Trim}. Conversely, {Mo_12_Trim} is the only ring from this family of clusters that incorporates a triply coordinated template (Figures 3 and S6).

Isophthalate templates promote the formation of the expanded {Mo_16_} host, [(Mo_2_O_2_Se_2_(OH)_2_)_8_(C_8_H_4_O_4_)_2_(H_2_O)_2_](NMe_4_)_4_, {Mo_16_(Isop)_2_} (Figure 3), in which two rigid aromatic guests are accommodated within the cavity. In this system, the ring adopts a pronounced elliptical distortion, indicating that template geometry is translated directly into host shape. In marked contrast, trimesate, bearing three carboxylate groups and a different connectivity and protonation profile, selectively stabilises a smaller {Mo_12_} ring, [(Mo_2_O_2_Se_2_(OH)_2_)_6_(C_9_O_6_H_4_)](NMe_4_)_2_, {Mo_12_Trim} (Figure 3), and remains bound over a wider pH window. The contrasting behaviour of Isop and Trim indicates that template‐directed nuclearity is influenced by more than the formal charge of the organic guest. Although both ligands provide carboxylate binding sites, they differ in connectivity, spatial arrangement, and the number of available functional groups, while Trim also possesses an additional carboxylic acid group with a distinct protonation state under the reaction conditions. Under more acidic conditions, the difference in their formal charge states would be reduced, potentially diminishing the electrostatic contribution to their different assembly preferences. The resulting differences in charge distribution, hydrogen‐bonding capability, and geometric presentation of the carboxylate groups may therefore contribute collectively to the preferential stabilisation of different ring architectures. Together, these observations suggest that relatively subtle changes in template geometry, connectivity, and protonation state can redirect the assembly pathway, while the present examples do not by themselves establish a universal templating rule.

**FIGURE 3:**
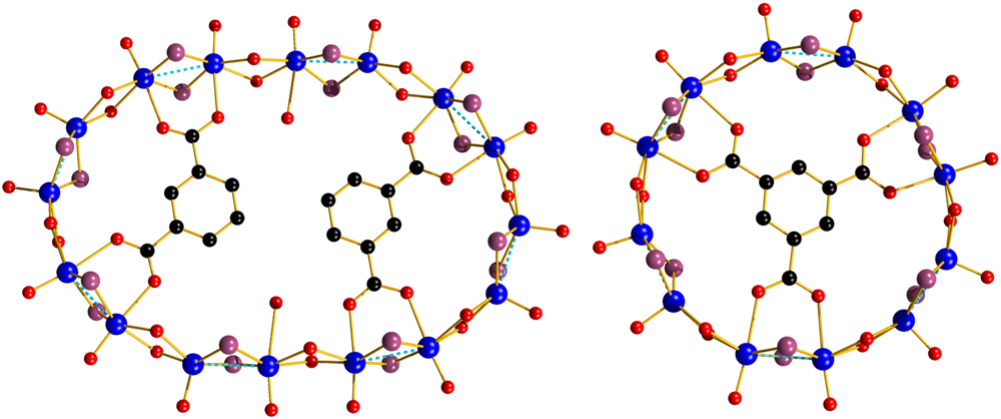
Ball‐and‐Stick representation of: {Mo_16_(Isop)_2_} (LEFT) and {Mo_12_Trim} (RIGHT). Colour code: Mo, blue; Se, purple; O, red; C, black; Mo– bonds, dashed light blue lines.

Squarate represents a distinct limiting case, combining high rigidity with strong hydrogen‐bonding capacity and compact geometry. Under optimised conditions, squarate directs the formation of the smallest ring in the templated series, [(Mo_2_O_2_Se_2_)_4_(OH)_6_(C_4_O_4_)(Mo^VI^O_4_H)_2_](NMe_4_)_2_, {Mo_8_(Mo^VI^)_2_(Sq)}, consistent with a contraction of the assembly pathway under strong geometric confinement. Together, these results demonstrate that aromatic templates act as programmable molecular inputs that write both nuclearity and ring distortion into the resulting Se‐based chalcoxide hosts. Notably, this level of template‐to‐host shape translation is consistent with increased electronic softness and conformational tolerance. Additionally, the pH dependence of the assembly also indicates that the reaction should be viewed as a dynamic speciation landscape rather than a sequence involving exclusively one discrete precursor at each stage. Within the intermediate pH regime, several partially condensed molybdenum‐selenium species may coexist in solution before the system evolves towards the thermodynamically favoured architectures under the corresponding reaction conditions. Thus, the absence of a crystallographically isolated intermediate should not be taken to imply its absence from solution. Rather, the experimentally isolated Mo_10_ and Mo_12_ products represent the reproducibly isolable architectures obtained under the respective optimised conditions.

These structural trends established the role of the template in tuning the nuclearity. Crystallographic characterisation (see Supporting Information) confirms that all members of this family share a common structural logic: edge‐sharing of [Mo_2_O_2_Se_2_]^2+^ dimers through bridging Se atoms. However, variation in ring size modulates Se coordination geometries, Mo‐Mo separations, and ring curvature, collectively reflecting the increased conformational freedom afforded by selenium. These features produce a distinctive structural hierarchy, and indicative of electronic flexibility that permeates the entire Se‐rich assembly. The structural diversity created by template control raised the question of whether these rings behave as static architectures or if they retain the dynamic flexibility suggested by their Se‐rich bonding. To probe the extent of this adaptability in solution, we investigated how the clusters respond to competing guest molecules using NMR titration. This allowed us to evaluate whether template binding is reversible and to what degree the ring frameworks accommodate external perturbations.

### 2.2 Dynamic Host–Guest Exchange versus Static Host–Guest System

Dynamic host–guest exchange in {Mo_12_Trim} and {Mo_16_Isop} was confirmed by NMR titration experiments (Figure 4). Incremental addition of competing ligands produced characteristic chemical‐shift changes and line broadening, consistent with the reversible exchange of encapsulated templates under mild conditions. These results establish the Se‐based rings as adaptive molecular hosts, capable of accommodating multiple guest species through coordination dynamics. The Se─Mo bonding network exhibits local flexibility, allowing reorganisation and re‐assembly. This adaptive host–guest behaviour represents a notable example of inorganic molecular dynamics comparable to classical organic supramolecular systems.

**FIGURE 4:**
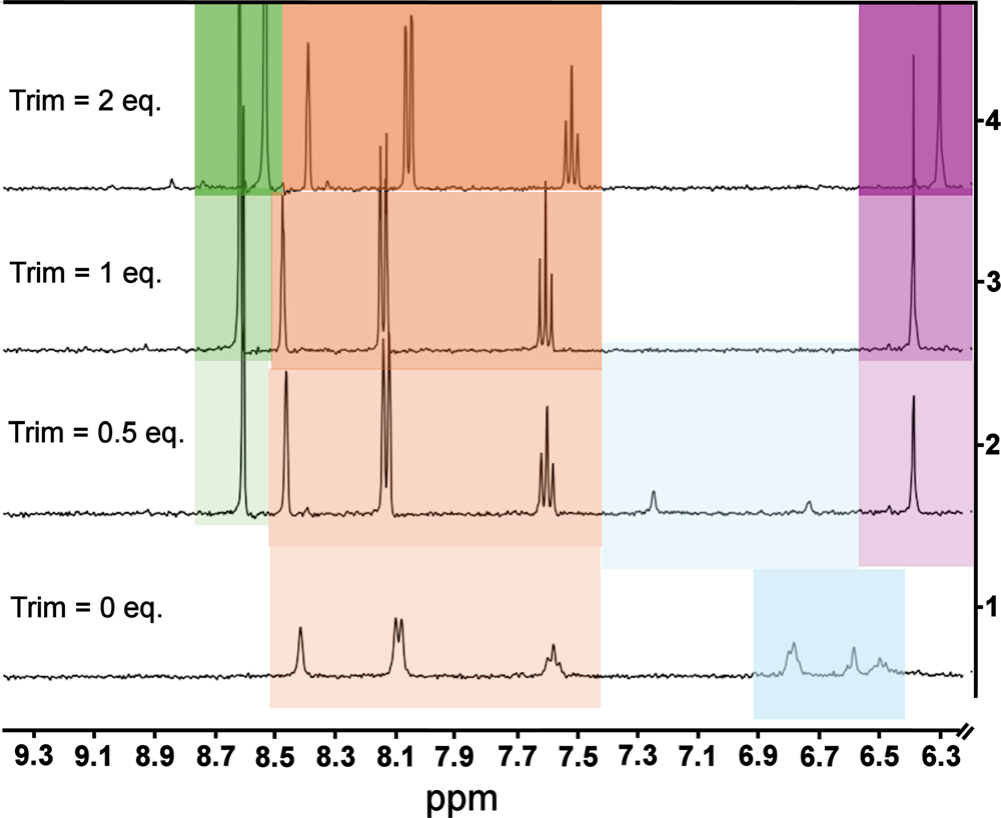
NMR titration of {Mo_16_(Isop)_2_} in D_2_O upon incremental addition of trimesate. Spectra shown from bottom to top: 0 µL (no ligand), 5 µL (0.5 equiv. trimesate, one ring), 10 µL (1 equiv. trimesate, one ring), and 20 µL (1 equiv. trimesate, two rings).

As discussed previously, valuable insight can be gained by examining our rings not only as bulk materials but as dynamically responsive host–guest systems in equilibrium with species present in aqueous solution. To investigate this behaviour, we carried out two titration experiments using aqueous solutions of preformed {Mo_16_(Isop)_2_} and {Mo_12_Trim}. Increasing amounts of isophthalate (added as isophthalic acid, pH ∼5) and trimesate (added as trimesic acid, pH ∼6) were introduced to each individual system, respectively. For both titrations, the molar ring: acid ratios employed were 1:0.5, 1:1, and 1:2. Additional NMR analyses, including all experimental details, are provided in the Supporting Information.

#### Titration of {Mo_16_(Isop)_2_} With Trimesate

The initial ^1^H NMR spectrum (no added ligand) matches the ^1^H NMR one (Figures S39 and S40). Six resonances are observed: three at 6.63, 6.71, and 6.91 ppm correspond to coordinated isophthalate (light‑blue area), while the higher‑field signals at 7.71, 8.21–8.23, and 8.55 ppm arise from free isophthalate (orange area). These features confirm the earlier analysis. Upon addition of trimesate at a 1:0.5 (ring:acid) ratio, the signals associated with coordinated isophthalate decrease markedly in intensity and shift downfield (to 6.73 and 7.25 ppm, light‑blue). A new singlet appears at 6.39 ppm, assigned to coordinated trimesate (purple rectangle), and a resonance for free trimesate emerges at 8.55 ppm (green rectangle). At 1:1 and 1:2 ratios, the resonances of coordinated isophthalate disappear entirely, while the trimesate signals at 6.39 and 8.55–8.62 ppm increase in intensity.

These observations establish the complete displacement of isophthalate from the cavity of the {Mo_16_} ring, with trimesate becoming the newly coordinated guest. Introduction of the trimesate guest generates a new chemical environment that triggers the contraction of the {Mo_16_} host into the smaller {Mo_12_} architecture, revealing a template‐induced reassembly process that demonstrates the adaptive character of the selenium‐based framework. Rather than undergoing simple guest substitution, the system reorganises through a co‐occurring structural rearrangement process in which the original {Mo_16_} ring adapts into the smaller {Mo_12_} host, demonstrating a template‐induced structural transformation driven by the intrinsic flexibility of the Se‐based framework.

#### ESI‐MS Studies

The NMR data alone do not allow us to determine unambiguously whether {Mo_16_(Isop)_2_} is converted into {Mo_12_(Trim)}, or whether excess trimesic acid induces formation of a potentially new species, for example, {Mo_x_(Trim)_y_}. ESI‐MS analysis of the reaction mixture provides the necessary clarification. While ^1^H.NMR confirms the presence of trimesate within the inner cavity, ESI‑MS distinguishes between {Mo_16_(Trim)_2_} and {Mo_12_Trim}. In this case, only the latter is observed. Two main isotopic distribution envelopes centred at 888 *m/z* (charge −3) and at 1370 *m/z* (charge −2), were observed respectively (Figure 5), which can be assigned to the fully converted host–guest species.

**FIGURE 5:**
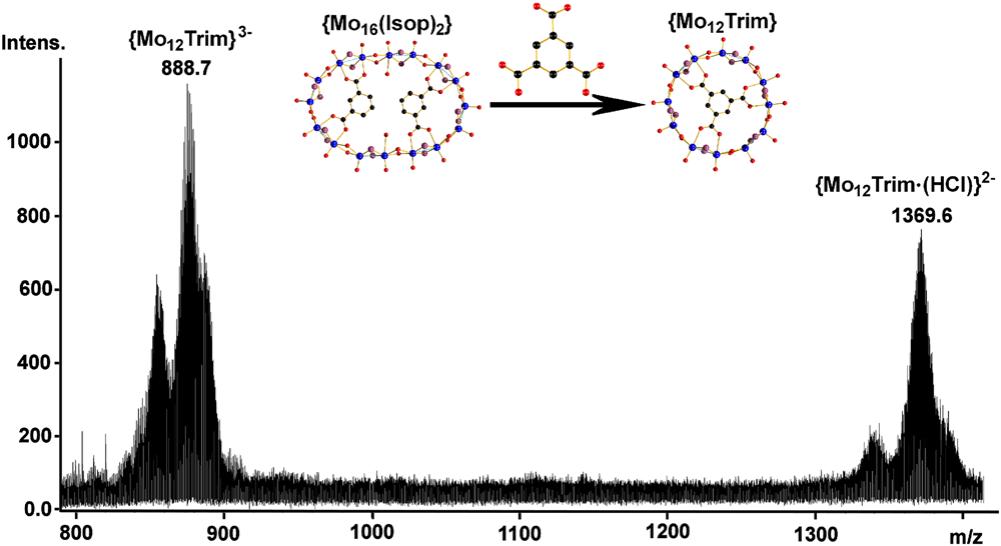
ESI‐MS spectrum demonstrating the complete structural rearrangement of {Mo_16_(Isop)_2_} in a solution containing 20 µL (1 equiv.) trimesic acid, showing doubly and triply charged isotopic envelopes of {Mo_12_Trim} as the final product.

#### Titration of {Mo_12_Trim} With Isophthalate

Upon addition of isophthalic acid to a D_2_O solution of {Mo_12_Trim}, no ligand exchange is observed. The system remains stable, and trimesate persists as the coordinated guest. Only signals corresponding to free isophthalic acid, free trimesate, and coordinated trimesate are detected, consistent with the earlier ^1^H NMR analysis (see SI). Taken together, the ^1^H NMR titrations and DOSY measurements demonstrate that trimesate forms a significantly more stable host–guest complex in aqueous solution than the isophthalate analogue (Figures S41‐S43).

The observation of dynamic response to environmental stimuli coupled with structural evolution demonstrates that these rings exhibit not only structural programmability but also functional adaptability. Such dynamic behaviour, often associated with electronic softness and accessible redox states, could potentially influence catalytic activity. Motivated by this correlation between structural flexibility and electronic function, we next examined whether nuclearity and chalcogen identity translate into measurable differences in electrocatalytic hydrogen evolution.

### 2.3 Electrocatalytic Hydrogen Evolution and Structure–Activity Correlation

The clusters were immobilised on glassy carbon electrode and evaluated for electrocatalytic hydrogen evolution under identical conditions, enabling direct comparison of their activity while minimising variations arising from electrode preparation and measurement protocols. A schematic overview of the electrode modification and electrochemical measurement procedure is provided in SI‐37. Comparison across the Se‐ring family reveals a clear nuclearity and electronic structure‐dependent catalytic trend (Mo_8_> Mo_12_> Mo_10_), indicating that the topology and electronic environment of the molybdenum framework directly influence the hydrogen evolution activity (Figures 6 and 7). Comparison with sulphur analogues shows that the Se family consistently outperforms its S‐based counterparts, exhibiting higher current densities and reduced overpotentials (Figures S50‐S54) [51, 52, 53]. The enhanced performance of the Se family is consistent with the greater polarizability and electronic softness of selenium, which may facilitate charge redistribution and proton‐coupled electron‐transfer processes across the cluster framework. The consistent structure‐activity correlation across both families indicates that nuclearity and chalcogen identity jointly define a nuclearity‐dependent catalytic landscape that parallels the structural programmability of the ring series. Experimentally, the catalysts exhibited optimal performance within the 0 to –0.5 V range versus NHE. Beyond –0.5 V, although the cathodic current increased substantially, the electrochemical signal became unstable, deviating from linearity (Supporting Information).

**FIGURE 6:**
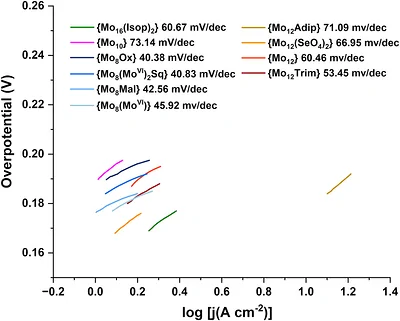
Tafel plots of the POSeM family.

**FIGURE 7:**
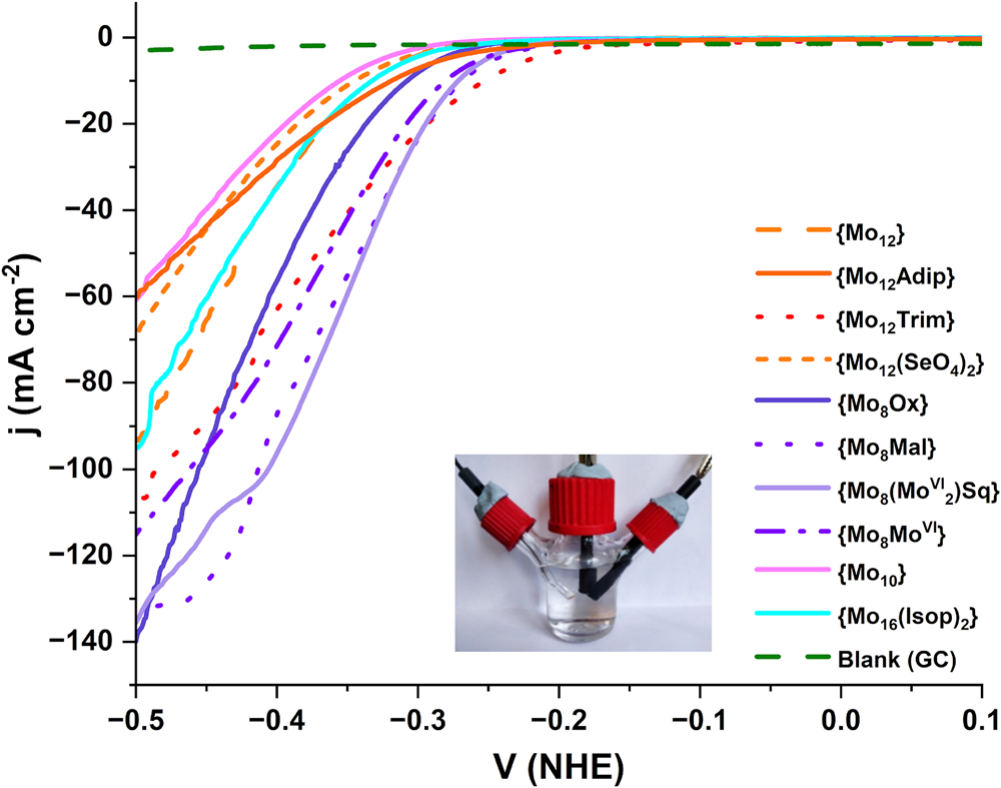
LSV measurements of POSeM rings in 1 M H_2_SO_4_. Inset: Custom‐made three‐electrode electrochemical cell used in this work. Glassy carbon electrode modified with different catalysts/loadings served as the working electrode, with a graphite rod as the counter electrode and an Ag│AgCl reference electrode.

The most promising activity was observed for the smallest family members, those with a nuclearity of 8 (violet curve in Figure 7). The species {Mo_8_Ox}, {Mo_8_Mal}, {Mo_8_(Mo^VI^)_2_(Sq)}, and {Mo_8_Mo^VI^} reached current densities of –140, –133, –135, and –115 mA cm^−2^ at –0.5 V, respectively. Interestingly, neither the ring size nor the identity of the templating agent appeared to provide a straightforward interpretation on the observed electrocatalytic behaviour. Under otherwise identical conditions, the three ring architectures exhibit clearly differentiated HER activity, following the order Mo_8_> Mo_12_> Mo_10_. This trend demonstrates that the catalytic response is not simply determined by the presence of the same Mo‐Se building units but is sensitive to their organisation within the molecular ring. The dependence of activity on nuclearity therefore provides a direct structure‐function relationship within this family of molecular chalcogenides.

For instance, {Mo_16_(Isop)_2_} outperformed both {Mo_10_} and {Mo_12_} rings. Likewise, {Mo_12_} showed enhanced HER activity relative to {Mo_12_Adip}, despite the latter being a guest‐free ring. These observations point toward a more complex catalytic mechanism than that established for simpler systems such as MoSe_2_ or {Mo_2_O_2_Se_6_}, where HER pathways are better understood and predominantly involve terminal selenium centres [53, 54, 55].

Further insight on the dynamics of the catalytic system is provided by the Tafel slope values, which fall between 40.38 and 73.14  mV dec–^1^. As expected, the best‐performing rings exhibit the lowest Tafel slopes, consistent with higher catalytic efficiency.

The substantial variability observed across onset potentials, Tafel slopes, and overpotentials suggests that each ring operates through a distinct and mechanistically distinguishable HER pathway. This is particularly striking given that all rings share the same fundamental building block, [Mo_2_O_2_Se_2_]^2+^, and that protonation is generally presumed to occur at selenium atoms [4, 55]. Thus, nuclearity not only defines structural topology but directly encodes catalytic behaviour, establishing a structure‐function continuum across the ring family.

The operational stability of the molecular catalysts was subsequently evaluated by consecutive cycling experiments using the same modified electrode. The catalysts were subjected to 500 consecutive electrochemical cycles at a current density of 20 mA cm^−2^ (Figures S59–S63). After 500 cycles, the potential required to sustain this current density changed by only 0.02–0.10 V, depending on the molecular cluster, indicating good retention of the electrocatalytic response under prolonged repeated operation. Importantly, the comparable stability observed across the family of ring architectures indicates that the differences in HER activity are not accompanied by substantial differences in their operational durability under the investigated conditions. To further assess the state of the molecular catalyst following prolonged electrochemical operation, the modified electrodes were examined by optical microscopy before and after the 500‐cycle experiments (Figure S71). No obvious macroscopic changes in the deposited material were observed. In addition, the deposited material was recovered by extraction with a compatible solvent and analysed by ESI‐MS (Figures S64–S70). The expected molecular ion corresponding to the respective POM was detected in the post‐electrolysis extract, providing evidence that the molecular cluster remains detectable following electrocatalytic operation. While these observations do not exclude transient structural or speciation changes under operating conditions, they support retention of the molecular POM following electrochemical treatment.

The observed divergence in catalytic behaviour points to a mechanistic landscape governed not only by Mo–Se connectivity but also by ring‐specific structure and electronic organisation. Together with the structural and host–guest studies, these results suggest that template‐directed nuclearity, adaptive behaviour and catalytic activity are interconnected consequences of the same Se‐based assembly landscape. This prompted us to examine the molecular origin of the catalytic differences computationally.

### 2.4 Computational Studies

The experimentally observed nuclearity dependence of HER activity prompted us to examine whether the different ring architectures also possess distinct energetic landscapes for the elementary steps of hydrogen evolution. We therefore employed density functional theory to investigate representative Mo_8_ and Mo_16_ selenium‐based rings and to identify the molecular features responsible for their different catalytic behaviour. More specifically, density functional theory (DFT) calculations [56, 57] were performed on representative {Mo_8_Ox} and {Mo_16_(Isop)_2_} using TPSSh‐D4 as a Kohn‐Sham Hamiltonian. The calculated free‐energy landscape indicates that hydrogen evolution proceeds through a heterolytic pathway involving sequential proton–electron transfer. A classical Volmer step was not identified for either cluster, as the strongly basic selenium atoms readily stabilise incoming protons, thereby favouring alternative proton‐coupled electron transfer pathways. Focus was therefore directed to bimolecular Tafel pathways and the thermodynamics of 2H^+^/2‐electron coupled processes were examined in both cases. It was revealed that the two most favourable protonation sites for {Mo_8_Ox} are located on the selenium bridge and on the terminal oxo moiety [*E* ^⦵^(2H^+^/2e– vs. SHE) = ‐1.59 V]. In the case of {Mo_16_(Isop)_2_} there are two sites to consider, one with the Trim ion coordinated (site A) and another with vacant coordination sites (site B), Figure S72. Bi‐reduction of site A will entail an equilibrium between the selenium + oxo sites [*E* ^⦵^(2H^+^/2e– vs. SHE) = ‐1.49 V] and the two oxo sites [*E* ^⦵^(2H^+^/2e– vs. SHE) = ‐1.48 V] which shows that despite the more accessible reduction potentials (100 mV) two different routes will be adopted for A sites.

Bi‐reduction of site B though will require a potential that is higher than in {Mo_8_Ox} [*E* ^⦵^(2H^+^/2e– vs. SHE) = ‐1.64 V] which is also in equilibrium with another protonated isomer that is not viable for hydrogen dissociation [*E* ^⦵^(2H^+^/2e– vs. SHE) = ‐1.67 V]. Using the most thermodynamically favoured isomers, the Tafel routes were evaluated (Figure 8) and their values compared. Both isomers of site A were included for comparison. Thus, site A of {Mo_16_(Isop)_2_} has a barrier height similar to that of {Mo_8_Ox}, but the former will also form another protonated isomer that will follow a kinetically much slower (*E*_a_ = 25 kcal mol–^1^) route in HER. Comparison of the two clusters further shows that the {Mo_8_} framework provides a more favourable energetic profile for hydrogen adsorption and subsequent H–H coupling than the larger {Mo_16_(Isop)_2_} ring, in agreement with the experimentally observed catalytic trend.

**FIGURE 8:**
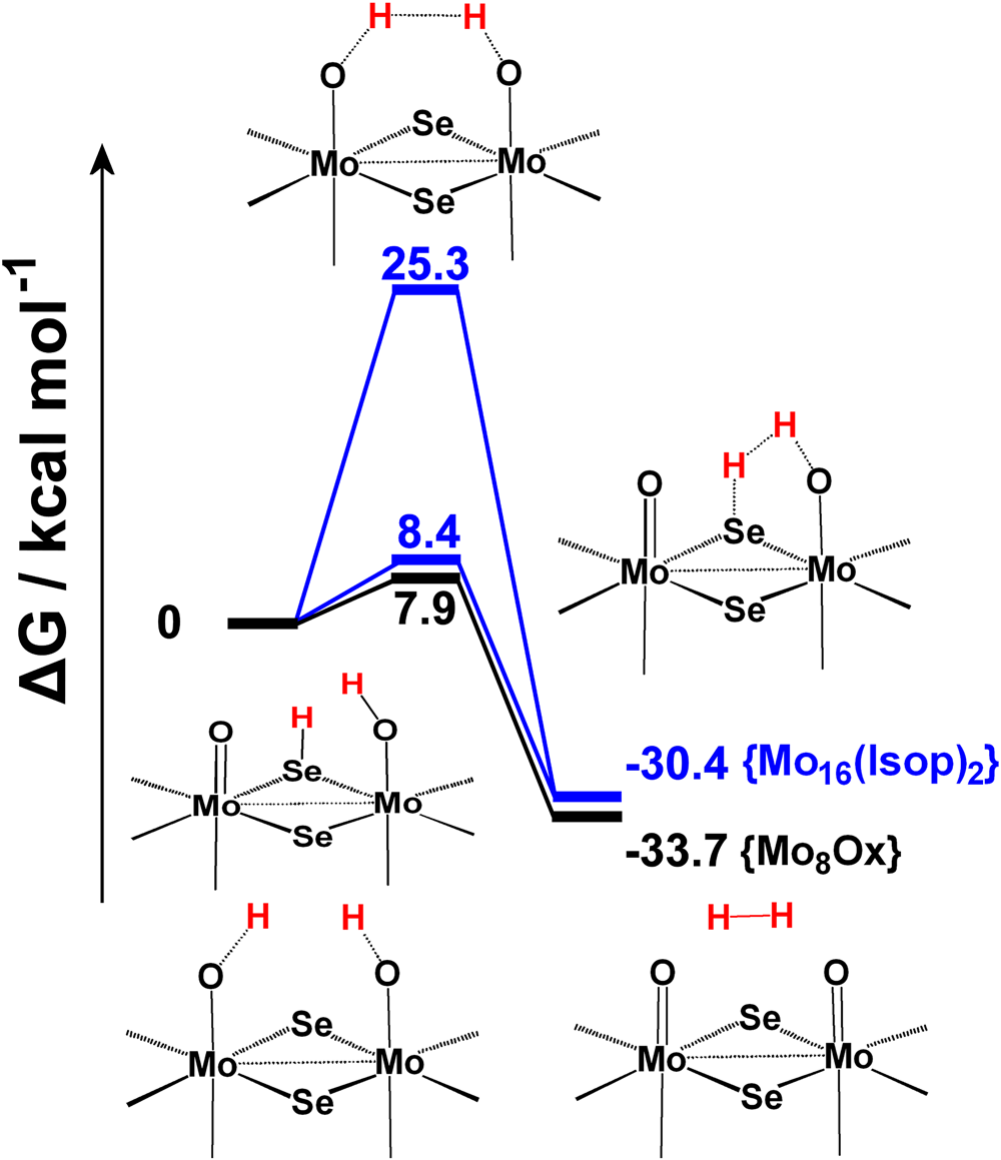
Calculated HER pathways for the doubly reduced species. Green pathways are for the {Mo_16_(Isop)_2_} species; the black one is the sole pathway of {Mo_8_Ox}.

These calculations therefore support the conclusion that subtle differences in cluster nuclearity and electronic structure modulate the accessibility of reactive selenium sites, linking the architecture of the chalcoxide ring directly to its catalytic behaviour.

### 2.5 Integrative Perspective

The combination of templated assembly, dynamic host–guest exchange, and catalytic function demonstrates a unified design principle for programmable inorganic chemistry. The [Mo_2_O_2_Se_2_]^2+^ dimer serves as both structural and functional unit, encoding nuclearity and reactivity through environmental inputs (template geometry, pH, and ligand substitution). The convergence of self‐assembly, adaptability, and activity marks an important step toward the rational construction of responsive inorganic materials. Together, these elements reveal a system in which molecular design principles operate across multiple hierarchical levels, from building blocks and templates to dynamics and function. This integrated behaviour positions the Se‐based chalcoxides as a platform for predictive inorganic design, setting the stage for broader implications and future developments.

## 3 Conclusions

We have established a general and programmable strategy for constructing selenium‐based molybdenum chalcoxides that unites templated self‐assembly, dynamic host–guest behaviour, and catalytic function within a single molecular platform. By using the [Mo_2_O_2_Se_2_]^2+^ dimer as a universal module, we demonstrate that nuclearity and topology can be deliberately encoded through template geometry, rigidity, and acidity, yielding a structurally diverse and rationally expandable family of rings spanning {Mo_8_Se_8_} to {Mo_16_Se_16_}.

Crucially, the incorporation of selenium imparts electronic softness and structural adaptability. This manifests in two notable behaviours: (i) adaptable guest exchange within the {Mo_16_Se_16_} host under mild conditions, accompanied by concomitant structural reorganisation to {Mo_12_Se_12_}, establishing a rare example of adaptive dynamics in a Se‐based inorganic ring system; and (ii) enhanced electrocatalytic hydrogen‐evolution activity following a clear structure‐activity trend ({Mo_8_} > {Mo_12_} > {Mo_10_}) that consistently outperforms sulphur‐based analogues.

Together, these findings define new design rules for chalcogenide cluster chemistry, linking structure, dynamics, and reactivity through a unified mechanistic framework. The modularity, responsiveness, and adaptability of this platform provide a blueprint for next‐generation functional inorganic materials, including adaptive catalysts, selective host–guest systems, and electronically tuneable molecular assemblies.

## Author Contributions

**Giulia Pellegrino**: investigation, validation, methodology, formal analysis, data curation, writing – original draft, visualization. **Alexander Elliott**: investigation, methodology, formal analysis, writing – review and editing, visualization. **Yu‐Fei Song**: investigation, methodology, writing – review and editing, formal analysis. **Nuno A. G. Bandeira**: investigation, methodology, software, data curation, visualization, writing – review and editing. **Haralampos N. Miras**: conceptualization, investigation, writing – original draft, writing – review and editing, visualization, methodology, validation, formal analysis, supervision, resources, project administration, funding acquisition.

## Conflicts of Interest

The authors declare no conflicts of interest.

## Supporting information



Data relevant to this study are provided in the Supporting Information file.
**Supporting File**: anie75036‐sup‐0001‐SuppMat.docx.

## Data Availability

The data that support the findings of this study are openly available in iochem‐BD repository at https://iochem‐bd.bsc.es/browse/review‐collection/100/494163/882a83ac8696bbffca6e4448. The optimised structures are available at the iochem‐BD repository [58, 59] at the following link: https://doi.org/10.19061/iochem‐bd‐6‐652.
