# Supplementary material for: Template‐Encoded Assembly and Adaptive Function in Selenium‐Based Molybdenum Chalcoxide Rings
Source: Angew Chem Int Ed Engl. 2026 Sep 29;65(46):e5129627. doi: 10.1002/anie.5129627 (PMC13749299; doi:10.1002/anie.5129627)
Supplement: Supplementary file 1 [file ANIE-65-e5129627-s001.docx]

**Supplementary Information**

**Template-Encoded Assembly and Adaptive Function in Selenium-Based Molybdenum Chalcoxide Rings**

Giulia Pellegrino,^[a]†^ Alexander Elliott,^[a]†^ Yu-Fei Song,*^[b]^ Nuno A. G. Bandeira,*^[c]^ and Haralampos N. Miras*^[a]^

**Table of contents**

1. **XRD	S2 - S6**
2. **PXRD	S7 - S12**
3. **INFRARED SPECTROSCOPY	S13 - S19**
4. **THERMAL ANALYSES	S20 - S25**
5. **NMR	S26 - S30**

**5.1**^1^H NMR/DOSY NMR

5.2 DOSY NMR
5.3 Titration NMR

6. **ESI-MS	S31 - S37**
7. **ELECTROCHEMISTRY	S37 - S49**

**7.1** Electrodes preparation

**7.2** LSV measurement

**7.3** Homogenous measurements

**7.4** Comparison with sulphur analogues

**7.5** Other relevant graphs

**7.6** Stability

8. **SYNTHETIC PROCEDURES**  **S49 - S54**

8.1 Synthesis [NMe_4_]_2_[Mo_2_O_2_(µ-Se)_2_(Se_2_)_2_] ({Se_6_})

8.2 Synthesis of [Mo_2_O_2_(µ-Se)_2_]^2+^

8.3 Untemplated Rings

8.4 Inorganic Rings

8.5 Organic Aliphatic Rings

8.6 Organic Aromatic Rings

9. **ELEMENTAL ANALYSES	S54**

**9.1** Analyses C,H,N

**9.2** Analyses Mo, Se

10. **DFT STUDIES	S55 - 64**
11. **REFERENCES** **S65**

1. **XRD**

Suitable single crystals were selected and mounted onto a rubber loop using Fomblin oil. Single-crystal datasets and unit cells were collected at 150(2) K on a Rigaku XtaLAB Synergy R HyPix-Arc diffractometer equipped with a graphite monochromator (λ_Mo-Kα_ = 0.71073 Å) on a microfocus X-ray source of rotating anode (50 kV, 24 mA). Data collection and reduction were performed using CrysAlisPro software package. Structure solution and refinement were carried out by using SHELXT-2018/3 and SHELXL-2018/3 ^[1]^ on WinGX platform.^[2]^ Most of the non-hydrogen atoms (including those disordered) were anisotropically refined. Corrections for incident and diffracted beam absorption effects were applied using analytical numeric absorption correction on multifaceted crystal models ^[3]^

**Figure S1**. Ball-and-Stick representation of **{Mo_8_Mo^VI^}**. Colour code: Mo, blue; Se, purple; O, red; Mo-Mo bonds, dashed light blue lines.

**Figure S2**. Structure of **{Mo_12_(SeO_4_)_2_}**. Colour code: Mo, blue; Se, purple; O, red ; H bonds, dashed blue lines.

**Figure S3.** Ball-and-Stick representation of **{Mo_8_Ox}**. Colour code: Mo, blue; Se, purple; O, red; C, black, H, white; Mo-Mo bonds, dashed light blue lines.

**Figure S4.** Ball-and-Stick representation of **{Mo_8_Mal}**. Colour code: Mo, blue; Se, purple; O, red; C, black; Mo-Mo bonds, dashed light blue lines**.**

.

**Figure S5**. Ball-and-Stick representation of **{Mo_12_Adip}**. Colour code: Mo, blue; Se, purple; O, red; C, black.

**Figure S6.** Ball-and-Stick representation of **{Mo_8_(Mo^VI^)_2_Sq}**. Colour code: Mo, blue; Se, purple; O, red; C, black; Mo-Mo bonds, dashed blue lines.

**Figure S7**. Ball-and-Stick representation of **{Mo_16_(Sq)_2_}**. Colour code: Mo, blue; Se, purple; O, red; C, black; Mo-Mo b, dashed blue light lines.

| **Compound** | **Mo-Mo (intra)** | **Mo-Mo (inter)** | **Mo=O** | **Mo-Chalcogen** | **Mo-O_av_** |
| --- | --- | --- | --- | --- | --- |
| {Mo_10_Se_10_ }  {Mo_10_S_10_ } ^[4]^ | 2.89(1)-2.92(2) | 3.31(1) | 1.67(8)-1.68(8) | 2.44(2)-2.45(1) | 2.54(8)-2.58(8) |
|  | 2.84(2)-2.85(2) | 3.32(2) | 1.66(1)-1.68(3) | 2.32(2)-2.33(2) | 2.57(4)-2.63(3) |
| {Mo_12_Se_12_} | 2.81(3)-2.82(2) | 3.3(3)-3.3(3) | 1.62(2)-1.72(1) | 2.40(2)-2.30(2) | 2.40(3)-2.90(6) |
| {Mo_12_S_12_} ^[5]^ | 2.80(1)-2.81(1) | 3.29(1)-3.32(1) | 1.67(1) | 2.29-2.33(4) | 2.59(1) |
| {Mo_12_Se_12_(SeO_4_)_2_} | 2.85(1)-2.93(3) | 3.19(1)-3.43(1) | 1.61(7) | 2.42(8)-2.45(8) | 2.05(7)-2.17(7) |
| {Mo_8_Mo^VI^Se_8_}  {Mo_8_Mo^VI^S_8_}^[6]^ | 2.82(1)-2.91(2) | 3.20(1) | 1.68(1)-1.71(1) | 2.46(2)-2.44(2) | 2.14(9)-2.07(8) |
|  | 2.84(6)-2.86(7) | 3.27(8) | 1.68(3)-1.70(5) | 2.32(5)-2.33(5) | 2.09(3)-2.15(4) |
| {Mo_8_Se_8_Ox}  {Mo_8_S_8_Ox} ^[7][8]^ | 2.86(4) | 3.2(4)-3.3(3) | 1.68(3) | 2.44(5)-2.45(4) | 2.08(3)-2.11(3) |
|  | 2.81(6) | 3.27(3) | 1.67(3)-1.69(3) | 2.32(5) | 2.081(2)-2.086(2) |
| {Mo_8_Se_8_ Mal} | 2.90(3) | 3.20(3) | 1.64(2)-1.69(2) | 2.44(6)-2.46(6) | 2.12(4) |
| {Mo_12_Se_12_Adip}  {Mo_12_S_12_Adip} ^[9][10]^ | 2.85(2)-2.90(1) | 3.26-3.42 | 1.63(1)-1.71(1) | 2.44(4)-2.46(4) | 2.04(4)-2.12(4) |
|  | 2.79(1)-2.84(1) | 3.23(2)-3.36(1) | 1.67(5)-1.71(4) | 2.33(1)-2.31(1) | 2.05(1)-2.12(1) |
| {Mo_8_(Mo^VI^)_2_Se_8_Sq}  {Mo_8_(Mo^VI^)_2_S_8_Sq} ^[11]^ | 2.8(2)-2.9(3) | 3.20(3) | 1.64(2)-1.67(2) | 2.43(4)-2.46(4) | 2.08(5)-2.12(5) |
|  | 2.8(1)-2.8(3) | 3.21(2) | 1.67(1)-1.69(1) | 2.31(4)-2.33(5) | 2.09(5)-2.12(5) |
| {Mo_16_Se_16_ (Isop)_2_}  {Mo_16_S_16_ (Isop)_2_}^[12]^ | 2.86(4)–2.90(4) | 3.31(6)–3.42(6) | 1.61(2)-1.77(3) | 2.42(4)-2.46(3) | 2.02(3)-2.09(3) |
|  | 2.8(1)-2.8(1) | 3.30(1)-3.40(1) | 1.68(7)-1.71(8) | 2.30(8)-2.33(9) | 2.05(1)-2.11(2) |
| {Mo_12_Se_12_Trim}  {Mo_12_S_12_Trim} ^[8]10]^ | 2.88(9)–2.89(6) | 3.37(8)-3.39(8) | 1.65(4)-1.71(5) | 2.45(4)-2.44(4) | 2.07(3)-2.08(4) |
|  | 2.82(1)-2.83(1) | 3.31(1)-3.41(1) | 1.67(6)-1.68(7) | 2.31(3)-2.33(2) | 2.05(5)-2.09(6) |

**Table S1.** Main bond distances.

**Table S2.** Ring dimensions.

|  | **Mo-Ox**  **Parallel (Å)** | **Mo-Ox**  **Perpendicular (Å)** | **Mo-Mal**  **Parallel (Å)** | **Mo-Mal**  **Perpendicular (Å)** |
| --- | --- | --- | --- | --- |
| **{Mo_8_Se_8_Ox}** | 2.48(3)-2.51(3) | 2.47(3) | – | – |
| **{Mo_8_S_8_Ox}^[7][8]^** | 2.49(3) | 2.5(3) | – | – |
| **{Mo_8_Se_8_Mal}** | – | – | 2.3(2)-2.3(2) | 2.4(2)-2.5(2) |

|  | **Mo-O_A_** | **Mo-O_B_** | **Mo-O_C_** | **Mo-O_D_** | **Long axis (L)** | **Short axis (S)** | **Difference (L) – (S)** |
| --- | --- | --- | --- | --- | --- | --- | --- |
| **{Mo_12_Se_12_Adip}** | 2.3(1) | 2.32(9) | 2.3 (1) | 2.3 (1) | 12.02 | 9.36 | 2.66 |
| **{Mo_12_S_12_Adip}^[9][10]^** | 2.30(1) | 2.33(1) | 2.27 (1) | 2.34 (1) | 11.80 | 8.44 | 3.36 |
|  |  |  |  | ***Difference Se vs Se** | *0.22 | *0.92 |  |

|  | **Mo-Isop (Å)** | **Isop-Isop (Å)** | **Mo-Trim (Å)** |
| --- | --- | --- | --- |
| **{Mo_16_Se_16_(Isop)_2_}** | 2.23(3)-2.42(3) | 3.59(6) | – |
| **{Mo_16_S_16_(Isop)_2_}^[12]^** | 2.26(7)-2.34(7) | 3.5(1) | – |
| **{Mo_12_Se_12_Trim}** | – | – | 2.32(5)-2.45(6) |
| **{Mo_12_S_12_Trim}^[8][10]^** | – | – | 2.35(9)-2.40(9) |

| Compound | O-C-O Angles (˚) | O-C-C Angles (˚) |
| --- | --- | --- |
| {Mo_8_Se_8_Ox}  {Mo_8_S_8_Ox}^[7][8]^ | α=β=125  α=β=133 | /  / |
| {Mo_8_Mal} | α=125, β=126 | / |
| {Mo_12_Se_12_Adip}  {Mo_12_S_12_Adip}^[9][10]^ | α=125, β=127  α=125, β=123 | /  / |
| {Mo_8_(Mo^VI^)_2_Sq} | / | α=131.36  β=136.50  γ=137.14  δ=134.89 |
| {Mo_16_Se_16_(Isop)_2_}  {Mo_16_S_16_(Isop)_2_}^[12]^ | α =115, β= 120, γ=131  δ=124  α =β =γ=δ=125 | /  / |
| {Mo_12_Se_12_Trim}  {Mo_12_S_12_Trim}^[8][10]^ | α =β =γ=124  α =β =γ=126 | /  / |

Table S3. Angles of carboxylate-templated rings.

2. **PXRD**

PXRD measurements were run on Miniflex from 3.000 (°) to 50.0000 (°) with a step of 0.1 (°) and a speed of 2.0 (°)/min with a Voltage of 40kV and current 15mA. The experimental data were systematically compared with simulations generated by the Mercury crystallographic software. The region between 5 and 10 degrees exhibits sharp peaks that are typical of compounds containing Mo-chalcogens. A review of the PXRD spectra from the {Mo_10_} experiments indicates a strong similarity between the spectra of Exp1 and Exp8, which supports the claims made in the synthetic section: despite the use of NH_4_Cl as a salt, a ring was formed with tetramethylammonium as the counterion.

**Figure S8**. PXRD of different experiments of **{Mo_10_}.**

**Figure S9**. Experimental (blue) and predicted (red) PXRD patterns of **{Mo_10_}** (Exp8).

**Figure S10**. Experimental (blue) and predicted (red) PXRD patterns of **{Mo_12_}.**

**Figure S11**: Experimental (blue) and predicted (red) PXRD patterns of **{Mo_8_Mo^VI^}**.

**Figure S12**. Experimental (blue) and predicted (red) PXRD patterns of **{Mo_12_(SeO_4_)_2_}.**

**Figure S13**.Experimental (blue) and predicted (red) PXRD patterns of **{Mo_8_Ox}.**

**Figure S14**. Experimental (blue) and predicted (red) PXRD patterns of **{Mo_8_Mal}.**

**Figure S16**. Experimental (blue) and predicted (red) PXRD patterns of **{Mo_16_(Isop)_2_}.**

**Figure S17**. Experimental (blue) and predicted (red) PXRD patterns of **{Mo_12_Trim}.**

3. **INFRARED SPECTROSCOPY**

FT-IR spectra were collected in transmission mode using a Nicolet iS5 FTIR Spectrometer, with 64 scans for each measurement. All spectra shown are recorded between 4000 - 500 cm^-1^.

All POSMs display infrared spectra characterized by a broad O-H signal between 3000-3500 cm^–1^ and a sharp signal in the vicinity of 1600-1620 cm^–1^, which corresponds to O-H stretching and H-O-H bending vibrations, respectively. Furthermore, for those POSMs containing tetramethylammonium as a counterion, an additional C-H bending signal is located at 1483 cm^–1^. It is worth noting that the tetramethyl ammonium cations could also be a remnant from prior synthetic steps. The molecular rings that accommodate organic templates containing carboxylate groups exhibit representative peaks centred at 1557 cm^–1^ and 1304 cm^–1^, which are associated with the asymmetric and symmetric stretches of the carboxylates. In the case of aromatic templates, such as trimesic, isophthalic, and squaric, the associated peaks may overlap with the C-C stretching vibrations of the benzene ring, which are located in the region of 1300 - 1600 cm^–1^, as well as at 1483 cm^–1^ when tetramethylammonium is also present. Additionally, in the same spectral region, aliphatic templates additionally contribute with the relevant bending vibrations of the C-H component of the aliphatic chains, such as in the case of malonate, oxalate, and adipate. Below 1000 cm^–1^, the signal observed in the region of 900 - 960 cm^–1^, is assigned to the Mo=O stretching vibration, along with bending vibrations of the Mo-O-Mo and Mo-Se-Mo populating the region between 500 - 800 cm^–1^.

For every experiment performed to produce **{Mo_10_}**, we recorded the infrared spectrum (Figure S18). The infrared data indicates the presence of TMA peaks centred at 1483 cm^–1^, with this region displaying peaks that are very similar to those found in the precursor [NMe_4_]_2_[Mo_2_O_2_(µ-Se)_2_(Se_2_)_2_. This finding corroborates the hypothesis outlined in the synthetic section concerning the role of NH_4_Cl, in addition to the results obtained from PXRD. In the case of **{Mo_12_}**, the observed peaks closely resemble those of **{Mo_10_}**, as anticipated. The presence of tetramethylammonium cations persists despite the absence of any anionic template, a finding corroborated by elemental analyses. Additionally, we identified a Mo^V^=O peak at slightly elevated wavenumbers (944 cm^–1^).

**Figure S18**. IR performed on different experiments of **{Mo_10_}.**

**Figure S19. (Exp8)** **{Mo_10_}** IR spectrum between 500 - 2000 cm^-1^ and 500 - 4000 cm^-1^ (inset). Peaks located at (cm^-1^) ~ 3000 (sh), 1614 (s), 1479 (s), 1442 (m), 1414 (m), 1287 (w), 1110 (w), 946 (s), s = strong, m = medium, w = weak, sh = shoulder.^[13][14]^

**Figure S20. {Mo_12_}** IR spectrum between 500 - 2000 cm^-1^ and 500 - 4000 cm^-1^ (inset). Peaks located at (cm^-1^) ~ 3000 (sh), 1613 (s), 1479 (s), 1442 (m), 1410 (m), 1285 (m), 1069 (m), 941 (s), 858 (m), 755 (sh), s = strong, m = medium, w = weak, sh = shoulder.^[13][14]^

**Figure S21. {Mo_8_Mo^VI^}** IR spectrum between 500 - 2000 cm^-1^ and 500 - 4000 cm^-1^ (inset). Peaks located at (cm^-1^) ~ 3000 (sh), 1607 (m), 1406 (w), 1070-1058 (sh), 939 (s), 813 (s), 725-711 (sh), s = strong, m = medium, w = weak, sh = shoulder.^[13][14]^

**Figure S22. {Mo_12_(SeO_4_)_2_}** IR spectrum between 500 - 2000 cm^-1^ and 500 - 4000 cm^-1^ (inset). Peaks located at (cm^-1^) ~ 3000 (sh), 1609 (m), 1059 (w), 943 (s), 813 (m), 729 (m). s = strong, m = medium, w = weak, sh = shoulder.^[13][14]^

**Figure S23. {Mo_8_Ox}** IR spectrum between 500 - 2000 cm^-1^ and 500 - 4000 cm^-1^ (inset). Peaks located at (cm^-1^) ~ 3000 (sh), 1835-1684 (w), 1558 (s), 1477 (m), 1301 (s), 929 (s), 780 (s), 630 (s), s = strong, m = medium, w = weak, sh = shoulder.^[13][14]^

**Figure S24. {Mo_8_Mal}** IR spectrum between 500 - 2000 cm^-1^ and 500 - 4000 cm^-1^ (inset). Peaks located at (cm^-1^) ~ 3000 (sh), 1552 (sh), 1403 (s), 1178 (sh), 938 (s), 820 (sh), 675-593 (sh), s = strong, m = medium, w = weak, sh = shoulder.^[13][14]^

**Figure S25.** **{Mo_12_Adip}** IR spectrum between 500 - 2000 cm^-1^ and 500 - 4000 cm^-1^ (inset). Peaks located at (cm^-1^) ~ 3000 (sh), 1614 (m), 1525 (m), 1480 (m), 1415 (sh), 949 (s), 809 (m), s = strong, m = medium, w = weak, sh = shoulder.^[13][14]^

**Figure S26.** **{Mo_8_(Mo^VI^)_2_Sq}** IR spectrum between 500 - 2000 cm^-1^ and 500 - 4000 cm^-1^ (inset). Peaks located at (cm^-1^) ~ 3000 (sh), 1615 (s), 1514 (s), 1480 (m), 1428 (sh), 1113 (m), 1067 (w), 939 (s), s = strong, m = medium, w = weak, sh = shoulder.^[13][14]^

**Figure S27.** **{Mo_16_(Isop)_2_}** IR spectrum between 500 - 2000 cm^-1^ and 500 - 4000 cm^-1^ (inset). Peaks located at (cm^-1^) ~ 3000 (sh), 1595 (s), 1537 (s), 1479 (s), 1441 (m), 1379 (s), 1271-1067 (w), 906 (s), 822 (w), 741 (m), 706 (s), 657 (m), 519-484 (sh), s = strong, m = medium, w = weak, sh = shoulder.^[13][14]^

**Figure S28. {Mo_12_Trim}** IR spectrum between 500 - 2000 cm^-1^ and 500 - 4000 cm^-1^ (inset). Peaks located at (cm^-1^) ~ 3000 (sh), 1597 (m), 1538 (m), 1479 (m), 1415 (m), 1360 (s), 1284 (w), 1099 (w), 915 (s), 758 (m), 717 (s), 689 (m), s = strong, m = medium, w = weak, sh = shoulder.^[13][14]^

4. **THERMAL ANALYSIS**

TGA were recorded on an SDT (simultaneous thermal analyser) Q600 instrument, manufactured by TA Instruments (A Division of Waters). Samples were run under inert atmosphere with a flux 100 ml/min Argon at a ramp rate of 10°C per minute, from 20° to 1000°.

For each POSM ring, we observed significant weight loss between 20 - 800°C. Specifically, from 20-100°C, the weight loss can be attributed to solvent content. The weight loss within the range 200 - 300 ^o^C, is associated to the removal of C and N content. Additionally, in the case of **{Mo_10_Se_10_}**, the loss of I_2_ is also noted in this temperature range, with a particularly intense derivative peak confirming the presence of I_2_ in the inner cavity, as corroborated by X-ray analysis and infrared spectroscopy. Furthermore, in the region of 300 ^o^C, we observe the loss of hydroxy bridges in the form of water. The experimental results validate the conclusions drawn from elemental analysis and X-ray diffraction. In the case of **{Mo_12_Se_12_}**, which was anticipated to be neutral and untemplated, we detected specific percentages of carbon and nitrogen attributable to Me_4_N^+^ salts precipitated from previous steps. Within the temperature range of 350-800^o^C the molecular rings exhibit a weight loss associated to the removal of the selenium content, mainly in the form of SeO_2_. In the final stages, sublimation of MoO_3_ occurs beyond 795^o^C. This observation agrees of the quantity of molybdenum that remains at temperatures exceeding 800 ^o^C, where the molybdenum content constitute approximately the 40% of the total molecular weight of the rings, which remains consistent beyond this temperature threshold.

**Figure S29.** Simultaneous thermal analysis (SDT) of **{Mo_10_}** presented as thermogravimetric (TGA) curves.

**Figure S30.** Simultaneous thermal analysis (SDT) of **{Mo_12_}** presented as thermogravimetric (TGA) curves.

**Figure S31.** Simultaneous thermal analysis (SDT) of **{Mo_8_Mo^VI^}** presented as thermogravimetric (TGA) curves.

**Figure S32.** Simultaneous thermal analysis (SDT) of **{Mo_12_(SeO_4_)_2_}** presented as thermogravimetric (TGA) curves.

**Figure S33.** Simultaneous thermal analysis (SDT) of **{Mo_8_Ox}** presented as thermogravimetric (TGA) curves.

**Figure S34.** Simultaneous thermal analysis (SDT) of **{Mo_8_Mal}** presented as thermogravimetric (TGA) curves.

**Figure S35.** Simultaneous thermal analysis (SDT) of **{Mo_12_Adip}** presented as thermogravimetric (TGA) curves.

**Figure S36.** Simultaneous thermal analysis (SDT) of **{Mo_8_(Mo^VI^)_2_Sq}** presented as thermogravimetric (TGA) curves.

**Figure S37.** Simultaneous thermal analysis (SDT) of **{Mo_16_(IsoP)_2_}** presented as thermogravimetric (TGA) curves.

**Figure S38.** Simultaneous thermal analysis (SDT) of **{Mo_12_Trim}** presented as thermogravimetric (TGA) curves.

5. **NMR**

NMR spectra were recorded at room temperature in D2O on Bruker Avance III HD 500 (500 MHz) or Avance III HD 400 (400 MHz) UltrashieldTM spectrometers. Every spectrum has been processed with baseline correction and phase correction.

**5.1 ^1^H NMR in D_2_O**

*Sample preparation*

Sample preparation for the NMR investigation involved dissolving 2 mg of the solid compound in 0.6 mL of 0.1 M NaOAc/AcOH buffer (pH 5.0) prepared in D₂O. NMR spectroscopy was employed to probe the solution-state behaviour of the species, particularly with respect to their relative stability and ring conformations. For **{Mo_12_Trim}**, a high-intensity singlet at δ = 6.30 ppm is observed, corresponding to three aromatic protons. In the case of **{Mo_16_(Isop)_2_}**, resonances attributable to both the free and the complexed guest can be distinguished in the aromatic region. The free guest exhibits a singlet at δ = 8.13 ppm (1H), doublets at δ = 8.12 ppm (2H), and a triplet at δ = 7.61 ppm (2H), consistent with protons of the benzene ring. Signals assigned to the complexed form are shifted upfield and comprise a singlet at δ = 6.80 ppm (1H), two low-intensity doublets in the region δ = 6.50–6.25 ppm (2H), and a singlet at δ = 5.91 ppm (1H), reflecting the altered electronic environment upon encapsulation.

**Figure S39.** ^1^H NMR of **{Mo_16_(IsoP)_2_}** in D_2_O; light green circled peaks correspond to the free isophthalic acid, while light blue circled peak to the complexed one.

**Figure S40.** ^1^H NMR of **{Mo_12_Trim}** in D_2_O; light blue circled peak corresponds to the complexed trimesic acid.

**5.2 DOSY NMR in D_2_O**

Diffusion-ordered spectroscopy (DOSY) was employed to investigate the behaviour of the rings in aqueous solution. Upon dissolution in water, the template-coordinated rings undergo partial hydrolysis, establishing an equilibrium between complexed and free species. DOSY measurements enable analysis of these equilibria by correlating chemical shifts with diffusion coefficients (D).

As expected, species of higher molecular weight exhibit lower diffusion coefficients, whereas smaller molecules diffuse more rapidly in solution. The highest diffusion coefficient is observed for acetate (buffer component), resonating at δ = 1.980–1.981 ppm, with D = (5.5–5.6) × 10^–9^ m^2^ s^–1^. The free tetramethylammonium counterion displays diffusion coefficients in the range D = (4.3–4.5) × 10^–9^ m^2^ s^–1^. For the macrocyclic hosts **{Mo_16_(Isop)_2_}** and **{Mo_12_Trim}**, diffusion coefficients of D = (1.0–1.5) × 10^–9^ m^2^ s^–1^ are associated with the complexed species, whereas values in the range D = (2.0–2.5) × 10^–9^ m^2^ s^–1^ correspond to the free guests. In the case of **{Mo_16_(Isop)_2_}**, six resonances were identified in the aromatic region: δ = 8.30 ppm (D = 2.11 × 10^–9^ m^2^ s^–1^), 7.99 ppm (2.28 × 10^–9^ m^2^ s^–1^), 7.97 ppm (2.27 × 10^–9^ m^2^ s^–1^), 7.50 ppm (2.31 × 10^–9^ m^2^ s^–1^), 7.48 ppm (2.27 × 10^–9^ m^2^ s^–1^), and 7.46 ppm (2.13 × 10^–9^ m^2^ s^–1^). On the basis of their chemical shifts and diffusion coefficients, these signals are assigned to the free acid. No resonances attributable to the complexed form were detected by DOSY. This observation is consistent with the corresponding ¹H NMR spectrum, in which the signals assigned to the encapsulated species are present only with very low intensity, falling below the detection threshold in the diffusion experiment. For the **{Mo_12_Trim}**, a resonance at δ = 6.301 ppm is associated with D = 1.17 × 10^–9^ m^2^ s^–1^, consistent with assignment to the complexed form. No signals corresponding to free trimesate (expected at δ ≈ 8.2 ppm) were observed. Although partial or complete hydrolysis is generally observed in these host–guest systems, in this case the proportion of free guest is likely too low to generate a detectable signal in the aromatic region. This is consistent with the dynamic guest exchange studies that are discussed in this work and confirmed by ESI-MS (Sample preparation was identical to that described in Section 5.1.).

**Figure S41.** DOSY NMR of **{Mo_16_(Isop)_2_}** in tetramethylammonium acetate buffer/D_2_O.

**Figure S42.** DOSY NMR of **{Mo_12_Trim}** in tetramethylammonium acetate buffer/D_2_O.

5.3 **NMR TITRATION IN D_2_O**

*Samples preparation*

**Vial 1**: a D_2_O solution of **{Mo_16_(Isop)_2_}** was prepared at a concentration of 2.11 mM

**Vial 2**: a D_2_O solution of **{Mo_12_Trim}** was prepared at a concentration of 2.92 mM

**Solution 3:** A 0.1 M solution of isophthalic acid was added to a 0.2 M solution of anhydrous LiOH in D₂O

**Solution 4:** A 0.1 M solution of trimesic acid was added to a 0.2 M solution of anhydrous LiOH in D₂O

The use of excess base ensured complete deprotonation of the acids, yielding the corresponding isophthalate and trimesate species. The resulting pH values were approximately 5.0 for **{Mo_16_(Isop)_2_}** and 5.5-6 for **{Mo_12_Trim}**.

Incremental additions were carried out as follows:

- To **Vial 1**, increasing volumes of **Solution 4** (trimesate) were added (5, 10, and 20 μL).
- To **Vial 2**, increasing volumes of **Solution 3** (isophthalate) were added (9, 18, and 36 μL).

*NMR analyses*

Prior to the addition of any ligand solution, a reference ¹H.NMR spectrum was recorded for each ring solution (**Vials 1** and **2**; 0 μL addition). Subsequently, eight ¹H.NMR spectra were acquired following each incremental ligand addition listed above.

**Figure S43.** NMR titration of **{Mo_12_Trim}** in D_2_O upon incremental addition of isophthalate. Spectra are shown from bottom to top: 0 μL (no ligand), 9 μL (isophthalate : ring = 0.5 : 1), 18 μL (isophthalate : ring = 1 : 1), and 36 μL (isophthalate : ring = 2 : 1).

6. **ESI-MS**

ESI-MS was performed using a Bruker MicrOTOF-Q quadrupole time-of-flight mass spectrometer. Samples were dissolved in CH_3_CN or CH_3_OH, introduced at a dry gas temperature of 180oC. The ion polarity for all MS scans recorded was negative, with the voltage of the capillary tip set at 4500 V, end plate offset at -500 V, funnel 1 RF at 400 Vpp and funnel 2 RF at 400 Vpp, hexapole RF at 200 Vpp, ion energy 5.0 eV, collision energy at 15 eV, collision cell RF at 1200 Vpp, transfer time at 120.0 µs, the pre-pulse storage time at 15.0 µs and analysed using the Bruker Daltonics v4.1 software.

Electrospray ionization mass spectrometry (ESI-MS) has been performed in CH_3_CN. The solution investigation by means of ESI-MS has been proven also in this case a very useful tool in extrapolating important structural information as well some indication on the self-assembly process related to the formation of our host-guests’ systems. The **{Mo_12_Adip}**, **{Mo_16_(Isop)_2_}** and **{Mo_12_(SeO_4_)_2_}** exhibit similar behaviour in solution. In the case of **{Mo_12_Adip}**, the observation of the singly charged distribution envelopes centred at 812.2, 852.0 and 885 m/z can be assigned fragments {(Mo_2_O_2_Se_2_)_2_O_2_OH)}^–^, {(Mo_2_O_2_Se_2_)_2_O_3_(CH_3_CN)}^–^ and {(Mo_2_O_2_Se_2_)_4_(OH)_4_O_2_ (C_6_H_8_O_4_)}^2–^ respectively. (Figure S47). The intact adipic-templated ring has been observed at higher m/z values with corresponding isotopic envelopes centred at 1338.4 and 1414.5 m/z, respectively. In the case of the isophthalic-templated host molecule a series of doubly charged distribution envelopes centred at 614.3, 679.3, 812 and 887.7 m/z values have been observed that can be assigned to {(Mo_2_O_2_Se_2_)_3_(OH)_3_O_2_}^2–^, {(Mo_2_O_2_Se_2_)_3_(OH)_6_(Me_4_NCl)}^2–^ and {(Mo_2_O_2_Se_2_)_3_(OH)_5_(C_8_H_4_O_4_)]^2–^, respectively. Similarly, the SeO_4_^2–^ templated ring, **{Mo_12_(SeO_4_)_2_}**, can be attributed to the envelope observed at 884.9 m/z and an associated fragment at 812 m/z assigned to {(Mo_2_O_2_Se_2_)_2_O_2_OH)}^–^.

Interestingly the Trimesate, Oxalate and Squarate templated rings **{Mo_12_Trim}**, **{Mo_8_Ox}** and **{Mo_8_(Mo^VI^)_2_Sq}** appear to be more robust under the experimental conditions. In the case of **{Mo_8_Ox}**, the doubly charged isotopic envelope centred at 875 m/z can be attributed to the intact ring {(Mo_2_O_2_Se_2_)(OH)_2_)_4_(C_2_O_4_)}^2–^. In the case of **{Mo_12_Trim}** two triply charged and a doubly charged envelopes with different combinations of counterions and solvent molecules, located at 888.7, 1064.7 and 1369.9 m/z can be attributed to the {(Mo_2_O_2_Se_2_)_6_(OH)_10_(C_9_H_4_O_6_)}^3–^, {(Mo_2_O_2_Se_2_)_6_(OH)_12_(C_9_H_4_O_6_)(CH_3_CN)_3_Cl_3_(Me_4_N)_3_}^3–^ and {(Mo_2_O_2_Se_2_)_6_(OH)_12_(C_9_H_4_O_6_)ClH}^2–^, respectively. Similarly, ESI-MS investigation of **{Mo_8_Mo^VI^}** retains its integrity and exhibits a series of envelopes with charges from -2 to -4 with different combinations of counterions, solvent molecules and fragments of the parent host-guest system (Figure S45). Moreover, the smaller member of the series **{Mo_8_Mo^VI^}** exhibits two doubly charge isotopic envelopes centred at 886.0 and 911.7 m/z which have been assigned to the intact ring-shaped structure.

**Figure S44.** ESI-MS of a) **{Mo_10_}** and b) **{Mo_12_}**.

The difference in the case of the **{Mo_12_}**, was mainly the solvent medium used due to solubility considerations (MeOH). Also in this case the intact ring has been observed at 1327.98 m/z {(Mo_2_Se_2_O_2_)_6_O_6_(OH)_2_(H_2_O)_6_(MeOH)_4_}^2–^ and at 1446.06 m/z {(Mo_2_Se_2_O_2_)_6_(OH)_12_(OH)_2_(H_2_O)_6_(MeOH)_8_}^2–^, as well to isotopic envelopes that can be attributed to the parent molecular structure centred at 2001.79, 1031.42 and 1210.89 m/z values assigned to {(Mo_2_Se_2_O_2_)_4_(OH)_9_(H_2_O)_9_(CH_3_OH)_5_}^–^ {(Mo_2_O_2_Se_2_)_2_(OH)_5_(H_2_O)_3_(CH_3_OH)_4_}^–^ and {(Mo_2_Se_2_O_2_)_3_O_3_(HO)}^–^ respectively. Finally, the intact **{Mo_8_Mal}** and **{Mo_10_}** exhibit doubly charged envelopes centred at 882.2 and 856.7 m/z values; for the malonate templated host ring {(Mo_2_O_2_Se_2_)_4_(OH)_8_C_3_H_2_O_4_}^2–^ and {(Mo_2_Se_2_O_2_)_4_O_5_(OH)_5_(H_2_O)_3_]^2–^; 1077 and 1020 m/z for the {Mo_10_} archetype host with {(Mo_2_O_2_Se_2_)_5_(OH)_10_(H_2_O)_4_}^2–^ and {(Mo_2_O_2_Se_2_)_5_O_8_H_4_}^2–^, respectively.

**Figure S45.** ESI-MS of c) **{Mo_8_Mo^VI^}** and d) **{Mo_12_(SeO_4_)_2_}**.

**Figure S46.** ESI-MS of e) **{Mo_8_Ox}** and f) **{Mo_8_Mal}**.

**Figure S47.** ESI-MS of **{Mo_12_Adip}**.

**Figure S48.** ESI-MS of **{Mo_8_(Mo^VI^)_2_Sq}**.

**Figure S49.** ESI-MS of g) **{Mo_16_(Isop)_2_}** and h) **{Mo_12_Trim}**.

|  | **Exp.** | **Theor.** | **Charge** | **Formula** |
| --- | --- | --- | --- | --- |
| **{Mo_10_}** | 1019.5 | 1020.5 | -2 | [(Mo_2_O_2_Se_2_)_5_(O)_8_H_4_] |
|  | 1077.9 | 1075.5 | -2 | [(Mo_2_O_2_Se_2_)_5_(OH)_10_(H_2_O)_4_] |
| **{Mo_12_}** | 1031.5 | 1030.8 | -1 | {(Mo_2_O_2_Se_2_)_2_(OH)_5_(H_2_O)_3_(CH_3_OH)_4_} |
|  | 1210.9 | 1210.4 | -1 | {(Mo_2_Se_2_O_2_)_3_(O)_3_(HO)} |
|  | 1327.9 | 1328.5 | -2 | {(Mo_2_Se_2_O_2_)_6_(O)_6_(HO)_2_(H_2_O)_6_(CH_3_OH)_4_} |
|  | 1446.0 | 1446.7 | -2 | {(Mo_2_Se_2_O_2_)_6_(OH)_12_(HO)_2_(H_2_O)_6_(CH_3_OH)_8_} |
|  | 2001.8 | 2002.6 | -1 | {(Mo_2_Se_2_O_2_)_4_(OH)_9_(H_2_O)_9_(CH_3_OH)_5_} |
| **{Mo_12_(SeO_4_)_2_}** | 812.0  884.9  1412.4  994.8 | 812.6  886.0  1411.8  994.6 | -1  -2  -2  -1 | {(Mo_2_O_2_Se_2_)_2_(O)_2_OH}  {(Mo_2_O_2_Se_2_)_4_(OH)_6_(SeO_4_)}  {[(Mo_2_O_2_Se_2_)_6_(OH)_12_(SeO_4_)_2_)H_2_](CH_3_CN)}  {(Mo_2_O_2_Se_2_)_2_(OH)_5_H_3_(SeO_4_)} |
| **{Mo_8_Mo^VI^}** | 911.7  886.0 | 911.6  885.7 | -2  -2 | {(Mo_2_O_2_Se_2_)_4_(OH)_8_)(MoO_4_)}  {[(Mo_2_O_2_Se_2_)_4_(OH)_8_)(OH)_2_ ]((CH_3_)_4_N)} |
| **{Mo_8_Ox}** | 875.2  856.7 | 875.6  857 | -2  -2 | {(Mo_2_O_2_Se_2_(OH)_2_)_4_C_2_O_4_)}  [((Mo_2_O_2_Se_2_)_4_(OH)_5_(O)_3_)(H_2_O)_3_] |
| **{Mo_8_Mal}** | 882.7 | 882.5 | -2 | [(Mo_2_O_2_Se_2_)_4_(OH)_8_(C_3_H_2_O_4_)] |
| **{Mo_12_Adip}** | 812.0  852.0  885.1  956.9  1211.6  1295.8  1414.5 | 812.6  852.6  885.7  957.3  1211.0  1296.4  1415.3 | -1  -1  -2  -2  -1  -2  -2 | {(Mo_2_O_2_Se_2_)_2_(O)_2_OH}  {(Mo_2_O_2_Se_2_)_2_(O)_3_ (CH_3_CN)}  {(Mo_2_O_2_Se_2_)_4_(OH)_4_(O_2_)C_6_H_8_O_4_}  {[(Mo_2_O_2_Se_2_)_4_(OH)_6_(H_2_O)C_6_H_8_O_4_](CH_3_CN)_3_}  {[(Mo_2_O_2_Se_2_)_2_(O)_3_(OH)C_6_H_8_O_4_](CH_3_CN)_4_((CH_3_)_4_N)}  {[(Mo_2_O_2_Se_2_)_5_(OH)_10_O_2_C_6_H_8_O_4_](CH_3_CN)((CH_3_)_4_N)_4_}  {[(Mo_2_O_2_Se_2_)_6_(OH)_12_C_6_H_8_O_4_](CH_3_CN)_2_((CH_3_)_4_N)Cl} |
| **{Mo_8_(Mo^VI^)_2_Sq}** | 750.2  820.0  870.0  914.0  1076.9  1383.0  1107.0 | 750.0  819.8  869.3  913.7  1077.1  1383.2  1106.8 | -4  -4  -3  -3  -2  -2  -3 | {[(Mo_2_O_2_Se_2_)_4_(OH)_6_(MoO_4_H)_2_C_2_O_4_][(Mo_2_O_2_Se_2_)_2_(OH)_4_(C_2_O_4_)](CH_3_CN)}  {[(Mo_2_O_2_Se_2_)_4_(OH)_6_(MoO_4_H)_2_C_2_O_4_] [(Mo_2_O_2_Se_2_)_2_(OH)_3_(C_2_O_4_)](CH_3_CN)((CH_3_)_4_N)_4_}  {[(Mo_2_O_2_Se_2_)_4_(OH)_6_(MoO_4_H)_2_C_2_O_4_][(Mo_2_O_2_Se_2_)(OH)C_2_O_4_](CH_3_CN)_2_}  {[(Mo_2_O_2_Se_2_)_4_(OH)_6_(MoO_4_H)_2_C_2_O_4_] (CH_3_CN)_4_(Me_4_N)}  ((Mo_2_O_2_Se_2_)_4_(OH)_6_(MoO_4_H)_2_C_2_O_4_)[(Mo_2_O_2_Se_2_)(OH)_4_C_2_O_4_](CH_3_CN)((CH_3_)_4_N)_2_  {[(Mo_2_O_2_Se_2_)_4_(OH)_6_(MoO_4_H)_2_C_2_O_4_][(Mo_2_O_2_Se_2_)_2_(OH)_3_C_2_O_4_](CH_3_CN)_2_((CH_3_)_4_N)_4_} |
| **{Mo_16_ (Isop)_2_}** | 614.3  679.3  887.6  960.1  1083.4 | 614.2  678.52  888.1  959.7  1084.1 | -2  -2  -2  -1  -2 | {(Mo_2_O_2_Se_2_)_3_(OH)_3_O_2_}  {[(Mo_2_O_2_Se_2_)_3_(OH)_6_Cl]((CH_3_)_4_N)}  {(Mo_2_O_2_Se_2_)_4_(OH)_5_C_8_H_4_O_4_}  {(Mo_2_O_2_Se_2_)_2_(OH)_2_(C_8_H_4_O_4_}  {[(Mo_2_O_2_Se_2_)_4_(O_H_)_10_C_8_H_4_O_4_](CH_3_CN)_3_((CH_3_)_4_N)_2_Cl} |
|  | 1212.7  1160.8  1083.4 | 1212.7  1159.6  1083.9 | -3  -2  -2 | {(Mo_2_O_2_Se_2_)_8_(OH)_15_(C_8_H_4_O_4_)_2_}  (Mo_2_O_2_Se_2_)_5_(OH)_10_(C_8_H_4_O_4_)(CH_3_CN)(H_2_O)_2_}  {(Mo_2_O_2_Se_2_)_4_(OH)_10_(C_8_H_4_O_4_)(CH_3_CN)_3_(Me_4_N)_2_Cl} |
| **{Mo_12_Trim}** | 1064.8  1369.6  888.7 | 1065.3  1369.9  889.6 | -3  -2  -3 | {[(Mo_2_O_2_Se_2_)_6_(OH)_12_(C_9_H_4_O_6_)(MeCN)_4_(Me_4_NCl)_3_]}  {(Mo_2_O_2_Se_2_)_8_(OH)_10_(C_9_H_4_O_6_)ClH}  {(Mo_2_O_2_Se_2_)_6_(OH)_10_C_9_H_4_O_6_} |

**Table S4.** Representation of the experimentally identified and simulated m/z values of POSM rings.

7. **Electrochemistry**

Electrochemical measurements were carried out using a CH Instruments CHI760D potentiostat in a custom-designed three-electrode electrochemical cell. The working electrode was a glassy carbon electrode (GCE) (BASi; diameter = 3.0 mm, geometric area = 0.071 cm²), with a graphite rod serving as the counter electrode and an Ag/AgCl (3 M KCl, BASi) electrode as the reference.

Prior to each measurement, the electrolyte solution and cell headspace were thoroughly purged with high-purity N_2_ gas for 20 minutes to remove dissolved oxygen. All reported potentials may be converted to the normal hydrogen electrode (NHE) scale using the relation: E(NHE) = E(Ag/AgCl) + 0.197 V.

All electrochemical data were corrected for iR drop to compensate for the uncompensated resistance of the electrolyte.

**7.1 Electrode preparation**

Working electrodes were fabricated by drop-casting and drying a catalytic ink onto the surface of a glassy carbon (GC) electrode. Prior to ink deposition, the electrode surface was mechanically polished to a mirror finish using an aqueous slurry of 0.05 μm alumina powder on a nylon polishing pad (Alvatek), followed by thorough rinsing with deionised water. Electrochemical cleaning was then performed via cyclic voltammetry (CV) in the potential range of 1.0 V to –1.2 V vs reference, for 30 consecutive cycles at a scan rate of 100 mV s⁻¹. Catalytic inks were formulated by dispersing the catalyst (ring) and carbon powder (Cabot, Vulcan X72R) in N,N-dimethylformamide (DMF), followed by the addition of 50 μL of Nafion solution, maintaining a mass ratio of ring to carbon of 2:1. Aliquots of 5 μL were drop-cast onto the polished GC electrode surface and allowed to dry at ambient temperature.

| **Compound** | **Carbon (mg)** | **Ring (mg)** | **DMF (μL)** | **Nafion (μL)** | **Drop cast volume (μL)** |
| --- | --- | --- | --- | --- | --- |
| **{Mo_10_}** | 5.1 | 5.3 | 500 | 50 | 5 |
| **{Mo_12_}** | 3.1 | 6.3 | 500 | 50 | 5 |
| **{Mo_12_(SeO_4_)_2_}** | 3 | 6 | 500 | 50 | 5 |
| **{Mo_8_Mo^VI^}** | 2.2 | 4.4 | 500 | 50 | 5 |
| **{Mo_8_Ox}** | 2 | 3.9 | 500 | 50 | 5 |
| **{Mo_8_Mal}** | 2 | 4 | 500 | 50 | 5 |
| **{Mo_12_Adip}** | 3 | 6.1 | 500 | 50 | 5 |
| **{Mo_8_(Mo^VI^)_2_Sq}** | 2.4 | 4.8 | 500 | 50 | 5 |
| **{Mo_16_(Isop)_2_}** | 4.7 | 9.5 | 1000 | 50 | 5 |
| **{Mo_12_Trim}** | 3.1 | 6.2 | 500 | 50 | 5 |

Table S5. catalysts loading on glassy carbon electrode surface.

**7.2 LSV (Linear Sweep Voltammetry) measurements**

A 1 M H₂SO₄ aqueous solution (pH ~ 0) was employed as the electrolyte. The working electrode consisted of a glassy carbon electrode (GCE) (BASi; diameter = 3.0 mm, geometric area = 0.071 cm^2^) onto which the catalyst under investigation had been deposited. A graphite rod was used as the counter electrode, and an Ag/AgCl reference electrode (3 M KCl, BASi) was employed. Polarisation curves were recorded using linear sweep voltammetry (LSV) at a scan rate of 5 mV s^–1^. All measurements were performed in triplicate. The potential window investigated ranged from 0 V to –1.0 V vs Ag/AgCl, (0.197 to –0.803 V vs NHE).

**Table S6**. Tafel slopes and potentials at 10, 20, and 50 mA cm^-2^ vs NHE for each chalcoxide ring.

| **Compound** | **Tafel Slope (mV/dec)** | **V at 10 mA cm^-2^ (NHE)** | **V at 20 mA cm^-2^ (NHE)** | **V at 50 mA cm^-2^ (NHE)** |
| --- | --- | --- | --- | --- |
| **{Mo_10_}** | 73.14 | -0.36 | -0.39 | -0.48 |
| **{Mo_12_}** | 60.46 | -0.33 | -0.37 | -0.43 |
| **{Mo_12_(SeO_4_)_2_}** | 66.95 | -0.34 | -0.39 | -0.46 |
| **{Mo_8_Mo^VI^}** | 45.92 | -0.28 | -0.31 | -0.36 |
| **{Mo_8_Ox}** | 40.38 | -0.31 | -0.34 | -0.39 |
| **{Mo_8_Mal}** | 42.56 | -0.27 | -0.29 | -0.34 |
| **{Mo_12_Adip}** | 71.09 | -0.32 | -0.37 | -0.47 |
| **{Mo_8_(Mo^VI^)_2_Sq}** | 40.83 | -0.27 | -0.29 | -0.34 |
| **{Mo_16_(Isop)_2_}** | 60.67 | -0.33 | -0.37 | -0.43 |
| **{Mo_12_Trim}** | 53.45 | -0.25 | -0.29 | -0.37 |

7.3 **Comparison with sulphur analogues**

To elucidate the influence of nuclearity on catalytic activity, we compared the selenium-based clusters **{Mo_10_Se_10_}**, **{Mo_12_Se_12_}**, and **{Mo_8_Se_8_Mo^VI^}** with their sulphur analogues **{Mo_10_S_10_}**, **{Mo_12_S_12_}**, and **{Mo_8_S_8_Mo^VI^}**, previously reported in the literature.^[4-6]^ Both the sulphur and selenium series display the same activity trend, with performance increasing in the order **{Mo_10_Se_10_}** < **{Mo_12_Se_12_}** < **{Mo_8_Se_8_Mo^VI^}.** An analogous sequence is observed for the corresponding sulphur clusters (Figure S50). This interpretation is further supported by analysis of the Tafel slopes (Figure S51). In addition to the linear sweep voltammetry (LSV) curves, the corresponding Tafel plots are presented, highlighting the systematic change in slope between the sulphur- and selenium-based rings. The two series differ by approximately 15–18 mV dec^–1^ (Figure S52), consistent with the trends observed in the LSV measurements. For quantitative comparison, the overpotentials required to reach current densities of 10, 20, and 50 mA cm^–2^ were extracted (Figure S53, Table S7). These values summarise the potential differences between the sulphur and selenium rings at defined catalytic current densities, providing a direct metric for evaluating the effect of chalcogen substitution and nuclearity on electrocatalytic performance.

**Figure S50.** LSV comparison of {Mo_10_Se_10_}, {Mo_12_Se_12_} and, {Mo_8_Se_8_Mo^VI^} with their sulphur analogues (0.3 - 0.5 V vs NHE). Colour code: {Mo_8_} light-blue, {Mo_10_} pink-lilac, {Mo_12_} orange-red.

**Figure S51.** Tafel slope comparison of **{Mo_10_Se_10_}**, **{Mo_12_Se_12_}** and, **{Mo_8_Se_8_Mo^VI^}** with their sulphur analogues (absolute overpotential on the x-axis; logarithm of the absolute current density on the y-axis).

**Table S7.** Tafel slopes and potentials at 10, 20, and 50 mA cm^-2^ vs NHE for **{Mo_10_Se_10_}**, **{Mo_12_Se_12_}** and, **{Mo_8_Se_8_Mo^VI^}** compared with their sulphur analogues.

| **Compound** | **Tafel Slope (mV/dec)** | **V at 10 mA** **cm^-2^ (NHE)** | **V at 20 mA** **cm^-2^ (NHE)** | **V at 50 mA** **cm^-2^ (NHE)** |
| --- | --- | --- | --- | --- |
| {Mo_10_Se_10_} | 73.14 | -0.36 | -0.39 | -0.48 |
| {Mo_10_S_10_} | 91.8 | -0.38 | -0.47 | / |
| {Mo_12_Se_12_} | 60.67 | -0.33 | -0.37 | -0.43 |
| {Mo_12_S_12_} | 76.4 | -0.30 | -0.34 | -0.47 |
| {Mo_8_Se_8_Mo^VI^} | 45.92 | -0.28 | -0.31 | -0.36 |
| {Mo_8_S_8_Mo^VI^} | 61.69 | -0.25 | -0.29 | -0.39 |

**Figure S52.** Difference in potential at 10, 20 and 50 mA cm^-2^, between **{Mo_10_Se_10_}**, **{Mo_12_Se_12_}**, **{Mo_8_Se_8_Mo^VI^}** and their sulphur analogues. Colour coding:{**Mo_8_}** blue, **{Mo_10_}** lilac-purple, **{Mo_12_}** red-orange-yellow.

**Figure S53.** Column plot showing the differences in Tafel slopes between **{Mo_10_Se_10_}**, **{Mo_12_Se_12_}**,**{Mo_8_Se_8_Mo^VI^}** and their sulphur analogues. Colour coding: **{Mo_8_}** light-blue, **{Mo_10_}** lilac, **{Mo_12_}** yellow.

Figure S54 (below) presents a column plot of the Tafel slope values for the oxothiomolybdate and oxoselenomolybdate rings. The values are displayed in descending order to emphasise the relative differences across the series. This visualisation clearly highlights the effect of nuclearity and chalcogen substitution on the electrocatalytic performance, allowing direct comparison between the sulphur and selenium-based rings.

**Figure S54.** Column plot showing differences in Tafel slope for the oxothiomolybdate and oxoselenomolybdate ring. Colour coding: **{Mo_8_}** violet-lilac, **{Mo_10_}** green, **{Mo_16_}** cyan and **{Mo_12_}** orange-red.

7.4 **Homogenous measurements**

To verify the absence of electrochemical activity under aprotic conditions, cyclic voltammetry (CV) was performed on a homogeneous solution comprising 2 mg of the ring dimer dissolved in 25 mL of acetonitrile, with 0.1 M tetrabutylammonium tetrafluoroborate (TBABF₄) as the supporting electrolyte. The same three-electrode configuration used for the heterogeneous measurements was employed, incorporating a clean, polished glassy carbon working electrode. CV measurements were conducted over a potential window ranging from 0 to –0.6 V vs Ag/AgCl at a scan rate of 100 mV s^–1^, with three consecutive cycles recorded.

**Figure S55.** Cyclic voltammogram of **{Mo_8_Mo^VI^}** recorded in CH_3_CN between 0.0 and −0.6 V vs. Ag/AgCl.

**Figure S56.** Cyclic voltammogram of **{Mo_8_Ox}** recorded in CH_3_CN between 0.0 and −0.6 V vs. Ag/AgCl.

**Figure S57.** Cyclic voltammogram of **{Mo_8_Mal}** recorded in CH_3_CN between 0.0 and −0.6 V vs. Ag/AgCl.

**Figure S58.** Cyclic voltammogram of **{Mo_8_(Mo^VI^)_2_Sq}** recorded in CH_3_CN between 0.0 and −0.6 V vs. Ag/AgCl.

7.6 **Electrochemistry and catalytic stability**

**Figure S59.** Polarization curve of **{Mo_8_Ox}** and **{Mo_8_Mal}** catalyst before (black line), after 100 (blue line) and after 500 cycles (red line). A graphite rod was used as counter electrode during stability measurement.

**Figure S60.** Polarization curve of **{Mo_8_Mo^VI^}** and **{Mo_8_(Mo^VI^_2_)Sq}** catalyst before (black line), after 100 (blue line) and after 500 cycles (red line). A graphite rod was used as counter electrode during stability measurement.

**Figure S61.** Polarization curve of **{Mo_10_}** and **{Mo_12_}** catalyst before (black line), after 100 (blue line) and after 500 cycles (red line). A graphite rod was used as counter electrode during stability measurement.

**Figure S62.** Polarization curve of **{Mo_12_Adip}** and **{Mo_12_(SeO_4_)_2_}** catalyst before (black line), after 100 (blue line) and after 500 cycles (red line). A graphite rod was used as counter electrode during stability measurement.

**Figure S63.** Polarization curve of **{Mo_12_Trim}** and **{Mo_16_Isop}** catalyst before (black line), after 100 (blue line) and after 500 cycles (red line). A graphite rod was used as counter electrode during stability measurement.

Additionally, after the catalytic procedure we extracted the molecular species in organic solvent and investigated again the speciation in the solution using high resolution electrospray ionization mass spectrometry. The ability to extract any molecular species present after the catalytic process and the observation of the same isotopic distribution envelopes in solution indicates that the species immobilized initially on the electrode retained their structural integrity.

**Figure S64.** ESI-MS spectrum of **{Mo_8_Ox}** and **{Mo_10_Mal}** before and after the catalytic process.

**Figure S65.** ESI-MS spectrum of **{Mo_9_}** before and after the catalytic process.

**Figure S66.** ESI-MS spectrum of **{Mo_12_Adip}** before and after the catalytic process.

**Figure S67.** ESI-MS spectrum of **{Mo_9_Sq}** before and after the catalytic process. Occasional intensity differences of the observed distribution envelopes is quite common specifically with large metal oxide species as reported in previous studies.^[11]^ Additionally, a small shift of some of the observed distribution envelopes is due to different number of solvent molecules associated with the species under investigation which can vary due different concentrations.

**Figure S68.** ESI-MS spectrum of **{Mo_12_Trim}** before and after the catalytic process.

**Figure S69.** ESI-MS spectrum of **{Mo_12_}** before and after the catalytic process.

**Figure S70.** ESI-MS spectrum of **{Mo_16_Isop}** before and after the catalytic process.

**Figure S71.** Optical microscopy photos of representative samples. The images show no obvious macroscopic changes to the deposited material following the catalytic measurements; a) **{Mo_12_}** and b) **{Mo_12_}**Trim before and after 500 cycles.

**8. SYNTHETIC PROCEDURES**

**8.1 Synthesis of [NMe_4_]_2_[Mo_2_O_2_(µ-Se)_2_(Se_2_)_2_] {Se_6_}** ^[15]^

Elemental selenium (4.736 g, 60 mmol) is suspended in aqueous 5% ammonium hydroxide (200 mL) and degassed by bubbling through nitrogen. The exhaust gas is passed through a bleach trap to destroy any hydrogen selenide produced. Sodium borohydride (472 mg, 12.5 mmol) in water (20 mL), degassed by bubbling through nitrogen, is added slowly to reduce the selenium, producing a dark red solution which is heated to 50°C for 30 minutes with stirring under a flow of nitrogen to ensure complete reduction. To this solution is then added a degassed solution of ammonium heptamolybdate (3.532 g,

20 mmol Mo^VI^) in water (50 mL) and the solution is then stirred overnight under a flow of nitrogen. Tetramethylammonium chloride (10 g, excess) is dissolved in water (20 mL) and added to the solution during which a dark brown solid rapidly precipitates [NMe_4_]_2_[Mo_2_O_2_(µ-Se)_2_(Se_2_)_2_ ({Se_6_}) which is collected by vacuum filtration and washed with cold water, cold ethanol, and diethyl ether.

(Yield: 65 %, based on Mo). Anal. Calcd.(%) for (C_10_H_27_N_3_O_2_Mo_2_Se_6_ Mr = 886.99): C, 13.54; H, 3.07; N, 4.74; Mo, 21.63. Found: C, 13.25; H, 2.92; N, 4.42; Mo, 22.02

A secondary step involves the oxidation reaction, carried out using iodine in an aqueous acidic solution, where two Se-Se bonds and four Mo-Se bonds are replaced by six water molecules, leading to the formation of the universal building blocks [Mo_2_O_2_Se_2_]^2+^, which appears as a dark red solution. Here below we report the synthetic route

**8.2 Synthesis of [Mo_2_O_2_(µ-Se)_2_]^2+^** ^[5]^

Crude [NMe_4_]_2_[Mo_2_O_2_(µ-Se)_2_(Se_2_)_2_ {Se_6_} (3.4 g, 4 mmol) is suspended in 40 mL H_2_O and degassed with bubbling nitrogen. A solution of I_3_^-^ was prepared by mixing elemental iodine (2.0 g, 8 mmol) with ammonium iodide (5 g, excess) in 60 mL 1M HCl followed by dropwise addition to the [NMe_4_]_2_[Mo_2_O_2_(µ-Se)_2_(Se_2_)_2_ {Se_6_} suspension under a flow of nitrogen. The solution was heated to 50°C and stirred for 15 minutes, then filtered whilst hot. The filtrate was cooled to 4°C and filtered a second time.^[27]^ (Yield 57%, based on Mo)

**8.3 Untemplated Rings**

**[(Mo_2_O_2_Se_2_(OH)_2_(H_2_O))_5_I_2_](NMe_4_)_2_ - {Mo_10_}**

To a filtered and degassed solution (under N_2_ atmosphere) of [Mo_2_O_2_(µ-Se)_2_]^2+^ (10 mL) lithium hydroxide (2 M) was added until it reaches a pH of 1.5. Ammonium chloride (1 g) was added. The resulting solution was stirred for 30 minutes under N_2_ atmosphere. The resulting solution was filtered and spit into several 5 mL portions fresh soda glass vials with Bakelite screw cap and allowed to stand for crystallisation in the fridge, for a week. Single crystals were isolated by filtration under vacuum and washed with cold ethanol and diethyl ether. (Yield: 20.90 %, based on Mo) Anal. Calcd.(%) for I_2_N_2_C_8_H_44_ O_25_Mo_10_Se_10_ (Mr = 2571.24): Mo, 37.17; Se, 30.71; C, 3.74; H, 1.72; N, 1.09. Found: Mo, 37.31; Se, 29.11; C, 4.01; H, 1.59; N, 0.96.

Table S8. {Mo_10_} experiments performed *(15° is the average temperature in our lab).

| Experiment | Salts added | pH | N_2_ | T (˚) |
| --- | --- | --- | --- | --- |
| 1 | TMACl_1 | 2.00 | no | 15 |
| 2 | KCl | 2.00 | no | 15 |
| 3 | CsCl | 2.00 | no | 15 |
| 4 | TMACl_2 | 1.50 | no | 15 |
| 5 | TMACl_3 | 1.50 | yes | 15 |
| 6 | TMACl_4 | 1.50 | yes | 4 |
| 7 | NH_4_Cl_1 | 1.50 | yes | 15 |
| 8 | NH_4_Cl_2 | 1.50 | yes | 4 |

**[(Mo_2_O_2_Se_2_(OH)_2_(H_2_O))_6_] {Mo_12_}**

10 mL of [Mo_2_O_2_(µ-Se)2]^2+^ solution, prepared as described earlier, was diluted by addition of 10 mL deionised water to minimise precipitation during the reaction. Aqueous 2 M LiOH was added dropwise with careful monitoring until the pH reached 5.0, during which the solution gradually darkened. Tetramethylammonium chloride (200 mg) was then introduced, and the mixture was stirred for 1 h to ensure complete dissolution and equilibration. The solution was filtered by gravity and split into several fresh 5 mL soda glass vials equipped with Bakelite screw caps. The vials were left undisturbed at room temperature for five days to allow crystallisation by slow evaporation. Single crystals formed during this period were isolated by vacuum filtration, washed sequentially with cold ethanol and diethyl ether, and dried under reduced pressure. (Yield: 24.50 %, based on Mo) Anal. Calcd.(%) for Cl_4_N_4_C_16_H_74_O_31_Mo_12_Se_12_ (Mr =3059.38): Mo, 37.63; Se, 30.97; C, 6.28; H, 2.44; N, 1.83. Found: Mo, 37.36, Se, 27.90; C, 7.03; H, 2.65; N, 1.71.

**8.4 Inorganic Rings**

**[(Mo_2_O_2_Se_2_(OH)_2_)_6_(SeO_4_)_2_(H_2_O)_2_]K_2_ {****Mo_12_(SeO_4_)_2_}**

10 mL of solution, 10 mL of [Mo_2_O_2_(µ-Se)_2_]^2+^ prepared as described earlier, was diluted by addition of 5 mL deionised water. A 4 mL aliquot of a 0.1 M solution of either glutaric acid or adipic acid was added to the reaction mixture; however, neither template was incorporated into the final crystal structure. Aqueous 2 M LiOH was then introduced dropwise to adjust the pH to 5.0, followed by the addition of potassium chloride (1.0 g). The resulting solution was stirred for 1h. Subsequently, the mixture was filtered by gravity and split into several fresh 5 mL soda glass vials fitted with Bakelite screw caps and left undisturbed at room temperature for ten days to enable crystallisation via slow evaporation. The crystals obtained were isolated by vacuum filtration, washed with cold ethanol and diethyl ether, and dried under reduced pressure. (Yield: 25.91 %, based on Mo) Anal. Calcd.(%) for K_2_H_16_O_34_Mo_12_Se_14_ (Mr = 2895.02): Mo, 39.77; H, 0.56. Found: Mo, 40.22; H, 0.43.

**[(Mo_2_O_2_Se_2_(OH)_2_)_4_Mo^VI^O_4_(H_2_O)_2_]K_2_** **{Mo_8_Mo^VI^}**

10 mL of [Mo_2_O_2_(µ-Se)_2_]^2+^ solution, prepared as described earlier, was diluted by addition of 10 mL deionised water. Sodium molybdate dihydrate (48 mg) was added to the solution and the solution was stirred. Cautious addition of 2 M lithium hydroxide follows to raise the pH of the solution to 5, followed by addition of potassium chloride (400 µL of a solution of 0.5 mg/10 mL). The solution was then stirred for 1 hour.

The resulting solution was filtered by gravity filtration into several 5 mL portions fresh soda glass vials with Bakelite screw cap and allowed to stand for crystallisation at RT, for three days. Crystals were precipitated out after slow evaporation process and filtered under vacuum. The precipitate was collected via vacuum filtration and washed with cold ethanol and diethyl ether. Crystals for x-ray diffraction are taken directly from the mother liquor.

An alternative way was synthetized **{Mo_10_}** without N_2_ supply, adding 1g of KCl instead of NH_4_Cl and recrystalise **{Mo_10_}** obtained in a minimum amount of water. (Yield: 52.20 %, based on Mo)

Anal. Calcd.(%) for K_2_H_12_O_22_Mo_9_Se_8_ (Mr =2125.03): Mo, 44.57; Se, 32.60; H, 0.57. Found: Mo, 44.41; Se, 31.41; H, 0.72.

**8.5 Organic Aliphatic Rings**

Solutions of templates are prepared at a concentration of 0.1 M in deionised water. Where the templates are not completely soluble in water, small portions of LiOH are added to deprotonate the template.

[(Mo_2_O_2_Se_2_(OH)_2_)_4_C_2_O_4_)](NMe_4_)_2_ **{Mo_8_Ox}**; [(Mo_2_O_2_Se_2_(OH)_2_)_4_C_3_H_2_O_4_)](NMe_4_)_2_[(Mo_2_O_2_Se_2_(OH)_2_)_4_ C_3_H_2_O_4_)](NMe_4_)_2_ **{Mo_8_Mal****}**; [(Mo_2_O_2_Se_2_(OH)_2_)_6_C_6_H_8_O_4_)](NMe_4_)_2_ **{Mo_12_Adip}**.

10 mL of [Mo_2_O_2_(µ-Se)_2_]^2+^ solution, prepared as described earlier, was diluted by addition of 10 mL deionised water. To this diluted solution was added 4 mL of 0.1 M template solution, and the mixture was stirred for 1 hour. Cautious addition of 2 M lithium hydroxide follows to raise the pH of the value of 5. The solution was then stirred for 1 hour. The resulting solution were filtered with gravity filtration into several 5 mL portions fresh soda glass vials with Bakelite screw cap and allowed to stand for crystallisation at RT, **{Mo_8_Ox}**,**{Mo_12_Adip}** for a week, **{Mo_8_Mal}** for three days). Crystals were precipitated out after slow evaporation process and filtered under vacuum. The precipitate was collected via vacuum filtration and washed with cold ethanol and diethyl ether. Crystals for x-ray diffraction are taken directly from the mother liquor.

**{Mo_8_Ox}** (Yield: 24.21 %, based on Mo)

Anal. calcd.(%) for (N_2_C_10_H_32_O_20_Mo_8_Se_8_ Mr =1899.56):

Mo, 40.40; Se,33.25; C, 6.30; H, 1.80; N, 1.47. Found: Mo, 40.20; Se, 32.18; C, 6.22; H, 1.59; N, 1.06.

{**Mo_8_Mal}**(Yield: 20.31 %, based on Mo) Anal. calcd.(%) for LiCl_2_N_3_C_7_H_30_O_20_Mo_8_Se_8_ (Mr = 1953.37):

Mo, 39.29; C, 4.30; H, 1.55; N, 2.15. Found: Mo, 39.26; C, 4.57; H, 1.36; N, 2.06.

**{Mo_12_Adip}**(Yield: 20.50 %, based on Mo) Anal. calcd.(%) for, ClN_3_C_18_H_68_O_34_Mo_12_Se_12_ (Mr = 3004.98):

Mo, 38.31 %; C, 7.19; H, 2.28; N,1.40. Found: Mo, 37.99, C, 6.63; H, 2.00; N, 1.46.

**8.6 Organic Aromatic Rings**

**[(Mo_2_O_2_Se_2_)_4_(OH)_6_(C_4_O_4_)(Mo^VI^O_4_H)_2_](NMe4)_2_****{Mo_8_(Mo^VI^)_2_Sq}**

The squaric acid (3,4-Dihydro xycyclobut-3-ene-1,2-dione) structure exhibited considerable difficulties in the isolation of Xray quality crystalline material. Unlike conventional organic templates, squaric acid was not a carboxylic acid, which may lead to strong ionic interactions with alkali metals during the deprotonation process that could partially obstruct the templated formation of a well-ordered crystalline structure. This results in a notably reduced coordination ability, as there are fewer nucleophile atoms available for coordination compared to the negatively charged oxygen atoms of carboxylic acids employed. This diminished coordination ability was also a result of its high acidity (it was the most acidic template moiety employed in this work. The increased conjugation within the organic ring correlates with its increased acidity, which subsequently reduces further the observed coordination ability.

During the course of our investigation, we succeeded in obtaining high-quality single crystals of **{Mo_8_(Mo^VI^)_2_(Sq)}**, by employing NH_4_OH instead of LiOH as a basic medium, while the pH maintained below the value of 4.30. Interestingly, we were also able to discover the presence of another structure under identical conditions, namely, [(Mo_2_O_2_Se_2_(OH)_2_)_8_(Sq)_2_(H_2_O)_2_]^4–^, **{Mo_16_(Sq)_2_}**. We hypothesized that the presence of O_2_ environment could favour the formation of **{Mo_8_(Mo^VI^)_2_Sq}**, due to the oxidative decomposition of the dimeric precursor, while under N_2_ atmosphere could lead to the formation of the **{Mo_16_(Sq)_2_}** ring species. Additionally, we attempted to favour further the formation of the Mo^VI^O_4_-containing moiety by adding a source of Mo(VI), specifically Na_2_MoO_4_. Unsurprisingly, addition of Na_2_MoO_4_ does not favour the formation of the **{Mo_8_(Mo^VI^)_2_Sq}** but instead leads to the formation of the more kinetically favourable species, **{Mo_8_Mo^VI^}** which highlights the presence of a plethora of dynamic equilibria that can be exploited and favour the formation of a desired moiety. The application of an inert atmosphere did not provide unambiguous proof of **{Mo_16_(Sq)_2_}** since we were not able to have reproducibility and enough product to conduct further analyses (Figure 1.7 ). As a result, it was apparent that our understanding of the combinatorial parameters that direct the formation of the **{Mo_16_(Sq)_2_}** ring was limited at this stage. Even though we were able to solve the structure of the isolated species, the data were not of sufficient quality and the relevant CIF file has been excluded at this stage.

**[(Mo_2_O_2_Se_2_)_4_(OH)_6_(C_4_O_4_)(Mo^VI^O_4_H)_2_](NMe4)_2_ {Mo_8_Mo^VI^Sq}**

10 mL of [Mo_2_O_2_(µ-Se)_2_]^2+^ solution, prepared as described earlier, was diluted by addition of 30 mL deionised water. 2 mL 0.1 M squaric acid (3,4-Dihydroxycyclobut-3-ene-1,2- dione) solution was added. Cautious addition of ammonia solution (40%) follows to raise the pH of the solution to 4.23. The solution was then stirred for 1 hour. The resulting solution were filtered with gravity filtration into several 5 mL portions fresh soda glass vials with Bakelite screw cap and allowed to stand for crystallisation at RT, for twenty days. Crystals were precipitated out after slow evaporation process and filtered under vacuum. The precipitate was collected via vacuum filtration and washed with cold ethanol and diethyl ether. Crystals for X-ray diffraction are taken directly from the mother liquor. (Yield: 20.09 %, based on Mo) Anal. calcd for N_2_C_12_H_32_O_26_Mo_10_Se_8_ (Mr =2337.01). Mo, 41.05; C, 6.17; H, 1.90; N,1.80. Found: Mo, 38.98; C, 5.74; H, 1.62; N, 1.67.

**Table S9.** **{Mo_8_Mo^VI^Sq}** experiments performed

| **N°** | **Ratio**  **Dimer/water** | **pH** | **Time**  **(minutes)** | **N_2_** | **Base** | **Solution Squaric Acid** | **Na_2_MoO_4_** |
| --- | --- | --- | --- | --- | --- | --- | --- |
| **1** | 1:3 | 5.00 | 30 | NO | LiOH | 0.1M | no |
| **2** | 1:3 | 4.92 | 30 | NO | LiOH | 0.1M | no |
| **3** | 1:3 | 4.88 | 60 | YES | NH_3_ | 0.1M | no |
| **4** | 1:4 | 4.92 | 30 | YES | NH_3_ | 0.1M | no |
| **5** | 1:5 | 4.91 | 30 | YES | NH_3_ | 0.1M | no |
| **6** | 1:1 | 6.5 | 10 | NO | KOH | Squaric* | no |
| **7** | 1:1 | 4.91 | 10 | NO | KOH | 0.1 M | no |
| **9** | 1:3 | 4.22 | 30 | NO | NH_3_ | 0.1 M | no |
| **10**  ****** | 1:3 | 4.23 | 60 | NO | LiOH | 0.25M | no |
| **11** | 1:3 | 4.24 | 60 | YES | LiOH | 0.25M | no |
| **13** | 1:3 | 4.26 | 60 | NO | NH_3_ | 0.25M | no |
| **14** | 1:3 | 4.28 | 60 | YES | NH_3_ | 0.25M | no |
| **15** | 1:3 | 4.24 | 60 | NO | NH_3_ | 0.1 M | YES |

* Squaric acid in solution dimer/water added without prior solubilized in water. (**from entry 10 we obtained also **{Mo_16_(Sq)_2_}** )

**[(Mo_2_O_2_Se_2_(OH)2)_8_(C_8_H_4_O_4_)_2_(H_2_O)_2_](NMe_4_)_4_** **{Mo_16_(IsoP)_2_}**

10 mL of 5 mL of [Mo_2_O_2_(µ-Se)_2_]^2+^ solution, prepared as described earlier, was diluted by addition of 10 mL deionised water. 4 mL 0.1 M isophthalic acid solution was added. Cautious addition of 2 M lithium hydroxide follows to raise the pH of the solution to 5. The solution was then stirred for 30 min. The resulting solution were filtered with gravity filtration into several 5 mL portions fresh soda glass vials with Bakelite screw cap and allowed to stand for crystallisation at RT, for a week. Crystals were precipitated out after slow evaporation process and filtered under vacuum. The precipitate was collected via vacuum filtration and washed with cold ethanol and diethyl ether. Crystals for x-ray diffraction are taken directly from the mother liquor. (Yield: 20.56 %, based on Mo) Anal. calcd for N_4_C_32_H_146_O_77_Mo_16_Se_16_ (Mr =4617.88): Mo, 33.24; C, 8.32; H, 3.18; N,1.21. Found: Mo, 33.00; C, 8.35; H, 3.02; N, 0.98.

**[(Mo_2_O_2_Se_2_(OH)_2_)_6_C_9_H_3_O_6_] (NMe_4_)_3_ {Mo_12_Trim}**

10 mL of [Mo_2_O_2_(µ-Se)_2_]^2+^ solution, prepared as described earlier, was diluted by the addition of 10 mL deionised water. 4 mL 0.1 M trimesic acid solution was added. Cautious addition of 2 M lithium hydroxide follows to raise the pH of the solution to 5.5. The solution was then stirred for 30 min. The resulting solution were filtered with gravity filtration into several 5 mL portions fresh soda glass vials with Bakelite screw cap and allowed to stand for crystallisation at RT, for a week. Crystals were precipitated out after slow evaporation process and filtered under vacuum. The precipitate was collected via vacuum filtration and washed with cold ethanol and diethyl ether. Crystals for x-ray diffraction are taken directly from the mother liquor. (Yield: 23.27 %, based on Mo) Anal. calcd for ClN_4_C_25_H_63_O_30_Mo_12_Se_12_ (Mr =3034.38): Mo, 38.19; C, 9.89; H, 2.09; N, 1.86. Found: Mo, 38.13; C, 8.40; H, 2.13; N, 2.04.

**9 ELEMENTAL ANALYSES**

**9.1 Analyses C,H,N**

Carbon, Hydrogen, and Nitrogen content of the synthesised Se-clusters were analysed on an Exeter CE-440 Elemental Analyser Version 10.03.21DDSA. The was conducted duplicates.

**9.2 Analyses Mo, Se**

Quantitative analysis of molybdenum (Mo) and selenium (Se) was conducted using an Agilent 5100 Inductively Coupled Plasma Optical Emission Spectrometer (ICP-OES), calibrated with commercially sourced Mo and Se standard solutions. Approximately 5 mg of each sample was digested in a solution comprising 2 mL of concentrated nitric acid (HNO_3_), 1 mL of deionised water, and 1 mL of hydrogen peroxide (H_2_O_2_). Following complete digestion, the containers were vigorously agitated to ensure oxidation of any hydrogen selenide (H_2_Se) potentially formed during the digestion process. Subsequently, ultrapure (ICP-grade) water was added carefully to each digest to bring the total volume to 50 mL.

**10. DFT studies**

**Computational Procedures and Methods**

Electronic structure calculations performed made use of the ORCA version 6.1 software^[17-19]^ package throughout. The hybrid TPSSh density functional^[20]^ was used in conjunction with Grimme’s D4 correction.^[21]^

The double-zeta def2-SV(P) basis set^[22]^ were used for the light elements and the def2-TZVP basis set^[23]^ with associated pseudo-potential^[24]^ for molybdenum.

The Resolution of Identity with the Chain of Spheres approximation^[25-30]^ (RIJCOSX) was used in the multi-centre integral solving with the default integration grid settings and corresponding auxiliary basis set^[31]^ (def2/J). Implicit solvation effects were modelled by employing the Conductor-like Polarizable Continuum Model^[32-34]^ (CPCM) with default settings for water. Analytic Hessians were computed for all the obtained stationary points to confirm their nature. Transition states underwent re-optimization with a fractional displacement of the imaginary vibrational mode to confirm the reaction pathway in both directions. Free Energies were computed for a temperature of 298.15 K and 1 atm pressure.

Absolute one-electron reduction potential of half-cell reactions were obtained from the free energies with the Nernst equation E_calc_(eV) = –ΔG° (eV) and the values subsequently shifted relative to the normal hydrogen electrode (NHE) with the experimental absolute reduction potential^[35]^ E*(NHE) = – 4.24 V such that E^Ꝋ^ (V)= E_calc_ - 4.24. The absolute free energy of a solvated proton was taken to be -11.53 eV as per the recommended guidelines^[36, 37]^

**Figure S72.** Depiction of distinct protonation sites **A** and **B** in **{Mo_16_(Isop)_2_}** ring.

The obtained crystallographic coordinates of **{Mo_8_Ox}** and **{Mo_16_(Isop)_2_}** were optimised with DFT at the TPSSh-D4 level. For **{Mo_8_Ox}** the total charge is –2 and the optimised Mo-Mo bond (2.827 Å, Figure S73) is in good agreement with the crystallographic one (2.861 Å) and the bridging Mo-Se is also quite close (2.450 vs. 2.445 Å). For **{Mo_16_(Isop)_2_}** the most relevant optimised bond lengths are also in good agreement with the experimental ones. In this structure however there are coordination vacancies in some of the Mo_2_^V^ bonded pairs which should make the electronic structure slightly distinct at the two unique sites. The ones which are coordinated to an anionic ligand shall be named sites A and the coordinatively vacant or aqua bound sites shall be named sites B in the ensuing discussion.

**Figure S73.** Optimised structures of **{Mo_8_Ox}** and **{Mo_16_(Isop)_2_}** with the most relevant bond lengths (Å).

The molecular orbitals are displayed on Figure S74. The electronic structure of **{Mo_8_Ox}** is that of a typical Mo(V) complex with six σ symmetric Mo-Mo single bonds. These bonds have a Mayer bond order of 0.73 and are localised solely within the selenium bridged pairs (Mo-Mo distance 2.8 Å). The hydroxo bridges impose a longer Mo-Mo distance (3.3 Å) and the Mo-Mo bond order is less than 0.01. The virtual MO set consists of the metal based π* orbitals with some admixing of the out-of-phase 2p orbitals from oxygen.

|  |  |  |
| --- | --- | --- |
| HOMO-7 (-6.29 eV) | HOMO-5 (-6.20 eV) | HOMO-4 (-6.20 eV) |
|  |  |  |
| HOMO-3 (-6.18 eV) | HOMO-2 (-6.14 eV) | HOMO-1 (-6.10 eV) |
|  |  |  |
| HOMO (-6.01 eV) | LUMO (-2.67 eV) | LUMO+1 (-2.60 eV) |

**Figure S74**. Kohn-Sham molecular orbital plots of {Mo_8_Ox}.

The electronic structure of **{Mo_16_(Isop)_2_}** is somewhat analogous (overall charge –4, Figure S75), i.e. the Mo(V) character with σ bonds filled but the LUMO instead of showing π(Mo-Mo) character shows a σ* antibonding set of orbitals at the vacant coordination site.

|  |  |
| --- | --- |
| **HOMO-10 (-6.13 eV)** | **HOMO-9 (-6.10 eV)** |
|  |  |
| **HOMO-8 (-6.09 eV)** | **HOMO-7 (-5.92 eV)** |
|  |  |
| **HOMO (-5.84 eV)** | **LUMO (-2.75 eV)** |
|  |  |
| **LUMO+1 (-2.75 eV)** | **LUMO+2 (-2.62 eV)** |

**Figure S75.** Kohn-Sham molecular orbital plots of **{Mo_16_(Isop)_2_}**.

However, the total density of states and oxygen projected counterparts show that the oxo groups in sites A are distinctive from those of sites B (Figure S77). Since the 2e^-^ reduction will occur at the metal centres this indicates that protons will more easily bind to the oxo groups in coordinatively saturated sites rather than the lower lying remaining oxo sites. This will be further discussed bellow when the proton coupled reductions are examined.

E(eV)

| Density of States | DOS |
| --- | --- |
| 5.4e-05 | -9 |
| 0.0001 | -8.997 |
| 0.000182 | -8.994 |
| 0.000326 | -8.991 |
| 0.00057 | -8.988 |
| 0.000979 | -8.98499 |
| 0.001648 | -8.98199 |
| 0.002721 | -8.97899 |
| 0.004406 | -8.97599 |
| 0.006997 | -8.97299 |
| 0.010895 | -8.96999 |
| 0.016638 | -8.96699 |
| 0.024917 | -8.96399 |
| 0.036597 | -8.96099 |
| 0.052714 | -8.95799 |
| 0.074465 | -8.95498 |
| 0.103165 | -8.95198 |
| 0.140174 | -8.94898 |
| 0.186795 | -8.94598 |
| 0.244133 | -8.94298 |
| 0.312937 | -8.93998 |
| 0.393424 | -8.93698 |
| 0.48511 | -8.93398 |
| 0.586678 | -8.93098 |
| 0.695893 | -8.92798 |
| 0.809601 | -8.92497 |
| 0.923822 | -8.92197 |
| 1.03394 | -8.91897 |
| 1.13501 | -8.91597 |
| 1.22209 | -8.91297 |
| 1.29064 | -8.90997 |
| 1.33696 | -8.90697 |
| 1.35846 | -8.90397 |
| 1.35397 | -8.90097 |
| 1.32382 | -8.89797 |
| 1.26984 | -8.89496 |
| 1.19522 | -8.89196 |
| 1.10421 | -8.88896 |
| 1.0018 | -8.88596 |
| 0.893333 | -8.88296 |
| 0.784127 | -8.87996 |
| 0.679158 | -8.87696 |
| 0.582803 | -8.87396 |
| 0.49868 | -8.87096 |
| 0.429574 | -8.86796 |
| 0.377431 | -8.86495 |
| 0.34341 | -8.86195 |
| 0.327959 | -8.85895 |
| 0.330903 | -8.85595 |
| 0.351517 | -8.85295 |
| 0.388587 | -8.84995 |
| 0.440452 | -8.84695 |
| 0.50503 | -8.84395 |
| 0.579852 | -8.84095 |
| 0.6621 | -8.83795 |
| 0.748664 | -8.83494 |
| 0.836231 | -8.83194 |
| 0.921389 | -8.82894 |
| 1.00076 | -8.82594 |
| 1.07116 | -8.82294 |
| 1.1297 | -8.81994 |
| 1.17397 | -8.81694 |
| 1.20215 | -8.81394 |
| 1.21308 | -8.81094 |
| 1.20633 | -8.80794 |
| 1.18223 | -8.80493 |
| 1.14189 | -8.80193 |
| 1.08708 | -8.79893 |
| 1.02021 | -8.79593 |
| 0.944185 | -8.79293 |
| 0.862299 | -8.78993 |
| 0.778046 | -8.78693 |
| 0.694976 | -8.78393 |
| 0.616535 | -8.78093 |
| 0.545915 | -8.77793 |
| 0.485923 | -8.77492 |
| 0.43888 | -8.77192 |
| 0.406539 | -8.76892 |
| 0.390035 | -8.76592 |
| 0.389861 | -8.76292 |
| 0.405868 | -8.75992 |
| 0.437293 | -8.75692 |
| 0.482809 | -8.75392 |
| 0.540605 | -8.75092 |
| 0.60849 | -8.74792 |
| 0.684018 | -8.74491 |
| 0.764645 | -8.74191 |
| 0.847876 | -8.73891 |
| 0.93143 | -8.73591 |
| 1.01338 | -8.73291 |
| 1.09224 | -8.72991 |
| 1.16708 | -8.72691 |
| 1.23747 | -8.72391 |
| 1.30347 | -8.72091 |
| 1.36555 | -8.71791 |
| 1.42435 | -8.7149 |
| 1.4806 | -8.7119 |
| 1.53485 | -8.7089 |
| 1.58729 | -8.7059 |
| 1.6376 | -8.7029 |
| 1.68486 | -8.6999 |
| 1.72748 | -8.6969 |
| 1.7633 | -8.6939 |
| 1.7897 | -8.6909 |
| 1.8039 | -8.6879 |
| 1.80321 | -8.68489 |
| 1.78541 | -8.68189 |
| 1.74904 | -8.67889 |
| 1.69374 | -8.67589 |
| 1.62044 | -8.67289 |
| 1.53143 | -8.66989 |
| 1.43035 | -8.66689 |
| 1.32201 | -8.66389 |
| 1.21215 | -8.66089 |
| 1.10707 | -8.65789 |
| 1.01322 | -8.65488 |
| 0.93684 | -8.65188 |
| 0.883552 | -8.64888 |
| 0.858016 | -8.64588 |
| 0.863647 | -8.64288 |
| 0.902403 | -8.63988 |
| 0.974625 | -8.63688 |
| 1.07897 | -8.63388 |
| 1.21239 | -8.63088 |
| 1.37027 | -8.62788 |
| 1.54656 | -8.62487 |
| 1.73409 | -8.62187 |
| 1.92492 | -8.61887 |
| 2.11085 | -8.61587 |
| 2.28391 | -8.61287 |
| 2.43688 | -8.60987 |
| 2.56382 | -8.60687 |
| 2.66051 | -8.60387 |
| 2.72468 | -8.60087 |
| 2.75618 | -8.59787 |
| 2.75688 | -8.59486 |
| 2.73039 | -8.59186 |
| 2.68167 | -8.58886 |
| 2.61643 | -8.58586 |
| 2.54058 | -8.58286 |
| 2.45962 | -8.57986 |
| 2.37821 | -8.57686 |
| 2.29985 | -8.57386 |
| 2.22674 | -8.57086 |
| 2.15989 | -8.56786 |
| 2.09936 | -8.56485 |
| 2.04466 | -8.56185 |
| 1.99514 | -8.55885 |
| 1.9504 | -8.55585 |
| 1.91056 | -8.55285 |
| 1.8764 | -8.54985 |
| 1.84927 | -8.54685 |
| 1.83092 | -8.54385 |
| 1.82307 | -8.54085 |
| 1.82704 | -8.53785 |
| 1.84331 | -8.53484 |
| 1.87123 | -8.53184 |
| 1.90881 | -8.52884 |
| 1.95278 | -8.52584 |
| 1.99878 | -8.52284 |
| 2.04174 | -8.51984 |
| 2.07641 | -8.51684 |
| 2.09789 | -8.51384 |
| 2.10216 | -8.51084 |
| 2.08653 | -8.50784 |
| 2.04989 | -8.50483 |
| 1.99285 | -8.50183 |
| 1.91762 | -8.49883 |
| 1.82777 | -8.49583 |
| 1.72782 | -8.49283 |
| 1.62275 | -8.48983 |
| 1.51751 | -8.48683 |
| 1.41658 | -8.48383 |
| 1.32354 | -8.48083 |
| 1.24087 | -8.47783 |
| 1.16986 | -8.47482 |
| 1.11064 | -8.47182 |
| 1.0624 | -8.46882 |
| 1.02364 | -8.46582 |
| 0.992538 | -8.46282 |
| 0.967257 | -8.45982 |
| 0.946247 | -8.45682 |
| 0.928434 | -8.45382 |
| 0.913312 | -8.45082 |
| 0.900928 | -8.44782 |
| 0.891761 | -8.44481 |
| 0.886542 | -8.44181 |
| 0.886048 | -8.43881 |
| 0.890902 | -8.43581 |
| 0.901432 | -8.43281 |
| 0.917599 | -8.42981 |
| 0.939008 | -8.42681 |
| 0.964993 | -8.42381 |
| 0.994754 | -8.42081 |
| 1.02752 | -8.41781 |
| 1.0627 | -8.4148 |
| 1.1 | -8.4118 |
| 1.13945 | -8.4088 |
| 1.18141 | -8.4058 |
| 1.22648 | -8.4028 |

| Density of States | O site A |
| --- | --- |
| 1e-06 | -9 |
| 2e-06 | -8.997 |
| 4e-06 | -8.994 |
| 8e-06 | -8.991 |
| 1.4e-05 | -8.988 |
| 2.4e-05 | -8.98499 |
| 4.1e-05 | -8.98199 |
| 6.8e-05 | -8.97899 |
| 0.00011 | -8.97599 |
| 0.000176 | -8.97299 |
| 0.000275 | -8.96999 |
| 0.000422 | -8.96699 |
| 0.000636 | -8.96399 |
| 0.000939 | -8.96099 |
| 0.00136 | -8.95799 |
| 0.001933 | -8.95498 |
| 0.002693 | -8.95198 |
| 0.003681 | -8.94898 |
| 0.004934 | -8.94598 |
| 0.006488 | -8.94298 |
| 0.008367 | -8.93998 |
| 0.010584 | -8.93698 |
| 0.013132 | -8.93398 |
| 0.01598 | -8.93098 |
| 0.019074 | -8.92798 |
| 0.022331 | -8.92497 |
| 0.025642 | -8.92197 |
| 0.028881 | -8.91897 |
| 0.031905 | -8.91597 |
| 0.03457 | -8.91297 |
| 0.03674 | -8.90997 |
| 0.038298 | -8.90697 |
| 0.039159 | -8.90397 |
| 0.039275 | -8.90097 |
| 0.038642 | -8.89797 |
| 0.0373 | -8.89496 |
| 0.035331 | -8.89196 |
| 0.032853 | -8.88896 |
| 0.030007 | -8.88596 |
| 0.026952 | -8.88296 |
| 0.023848 | -8.87996 |
| 0.020853 | -8.87696 |
| 0.018108 | -8.87396 |
| 0.015736 | -8.87096 |
| 0.01384 | -8.86796 |
| 0.012498 | -8.86495 |
| 0.011766 | -8.86195 |
| 0.01168 | -8.85895 |
| 0.012258 | -8.85595 |
| 0.013501 | -8.85295 |
| 0.015392 | -8.84995 |
| 0.017899 | -8.84695 |
| 0.020973 | -8.84395 |
| 0.024547 | -8.84095 |
| 0.028535 | -8.83795 |
| 0.032835 | -8.83494 |
| 0.037323 | -8.83194 |
| 0.041864 | -8.82894 |
| 0.046308 | -8.82594 |
| 0.050498 | -8.82294 |
| 0.054276 | -8.81994 |
| 0.057492 | -8.81694 |
| 0.060007 | -8.81394 |
| 0.061709 | -8.81094 |
| 0.062512 | -8.80794 |
| 0.062372 | -8.80493 |
| 0.061283 | -8.80193 |
| 0.059287 | -8.79893 |
| 0.056468 | -8.79593 |
| 0.052951 | -8.79293 |
| 0.048895 | -8.78993 |
| 0.04448 | -8.78693 |
| 0.039903 | -8.78393 |
| 0.035358 | -8.78093 |
| 0.031031 | -8.77793 |
| 0.027089 | -8.77492 |
| 0.023668 | -8.77192 |
| 0.020876 | -8.76892 |
| 0.01878 | -8.76592 |
| 0.017417 | -8.76292 |
| 0.016788 | -8.75992 |
| 0.016864 | -8.75692 |
| 0.017593 | -8.75392 |
| 0.018902 | -8.75092 |
| 0.020706 | -8.74792 |
| 0.022914 | -8.74491 |
| 0.025435 | -8.74191 |
| 0.028183 | -8.73891 |
| 0.031086 | -8.73591 |
| 0.034086 | -8.73291 |
| 0.037144 | -8.72991 |
| 0.040241 | -8.72691 |
| 0.043378 | -8.72391 |
| 0.046571 | -8.72091 |
| 0.049845 | -8.71791 |
| 0.053229 | -8.7149 |
| 0.056742 | -8.7119 |
| 0.060392 | -8.7089 |
| 0.064158 | -8.7059 |
| 0.067989 | -8.7029 |
| 0.071797 | -8.6999 |
| 0.075459 | -8.6969 |
| 0.078818 | -8.6939 |
| 0.081696 | -8.6909 |
| 0.083909 | -8.6879 |
| 0.085277 | -8.68489 |
| 0.085651 | -8.68189 |
| 0.084927 | -8.67889 |
| 0.083062 | -8.67589 |
| 0.080082 | -8.67289 |
| 0.07609 | -8.66989 |
| 0.071256 | -8.66689 |
| 0.065812 | -8.66389 |
| 0.060036 | -8.66089 |
| 0.054229 | -8.65789 |
| 0.048698 | -8.65488 |
| 0.043734 | -8.65188 |
| 0.039594 | -8.64888 |
| 0.036487 | -8.64588 |
| 0.034563 | -8.64288 |
| 0.033907 | -8.63988 |
| 0.034538 | -8.63688 |
| 0.036405 | -8.63388 |
| 0.039398 | -8.63088 |
| 0.043349 | -8.62788 |
| 0.048044 | -8.62487 |
| 0.053233 | -8.62187 |
| 0.058643 | -8.61887 |
| 0.063996 | -8.61587 |
| 0.069024 | -8.61287 |
| 0.073485 | -8.60987 |
| 0.077179 | -8.60687 |
| 0.07996 | -8.60387 |
| 0.081747 | -8.60087 |
| 0.082524 | -8.59787 |
| 0.082341 | -8.59486 |
| 0.081305 | -8.59186 |
| 0.079568 | -8.58886 |
| 0.07731 | -8.58586 |
| 0.074722 | -8.58286 |
| 0.071994 | -8.57986 |
| 0.069297 | -8.57686 |
| 0.066778 | -8.57386 |
| 0.064558 | -8.57086 |
| 0.062735 | -8.56786 |
| 0.061395 | -8.56485 |
| 0.060621 | -8.56185 |
| 0.060506 | -8.55885 |
| 0.061168 | -8.55585 |
| 0.062748 | -8.55285 |
| 0.065415 | -8.54985 |
| 0.069352 | -8.54685 |
| 0.074744 | -8.54385 |
| 0.081748 | -8.54085 |
| 0.090473 | -8.53785 |
| 0.100945 | -8.53484 |
| 0.11309 | -8.53184 |
| 0.126712 | -8.52884 |
| 0.141488 | -8.52584 |
| 0.156975 | -8.52284 |
| 0.172633 | -8.51984 |
| 0.187856 | -8.51684 |
| 0.202018 | -8.51384 |
| 0.214526 | -8.51084 |
| 0.224868 | -8.50784 |
| 0.232665 | -8.50483 |
| 0.237704 | -8.50183 |
| 0.23996 | -8.49883 |
| 0.239594 | -8.49583 |
| 0.236934 | -8.49283 |
| 0.232441 | -8.48983 |
| 0.22665 | -8.48683 |
| 0.22012 | -8.48383 |
| 0.213375 | -8.48083 |
| 0.206854 | -8.47783 |
| 0.200885 | -8.47482 |
| 0.19567 | -8.47182 |
| 0.191297 | -8.46882 |
| 0.18776 | -8.46582 |
| 0.185001 | -8.46282 |
| 0.182948 | -8.45982 |
| 0.181554 | -8.45682 |
| 0.180819 | -8.45382 |
| 0.180802 | -8.45082 |
| 0.181612 | -8.44782 |
| 0.183382 | -8.44481 |
| 0.186237 | -8.44181 |
| 0.19026 | -8.43881 |
| 0.195453 | -8.43581 |
| 0.201724 | -8.43281 |
| 0.208873 | -8.42981 |
| 0.216609 | -8.42681 |
| 0.22457 | -8.42381 |
| 0.232358 | -8.42081 |
| 0.23958 | -8.41781 |
| 0.245876 | -8.4148 |
| 0.250945 | -8.4118 |
| 0.254562 | -8.4088 |
| 0.256571 | -8.4058 |
| 0.256884 | -8.4028 |

| Density of States | O site B |
| --- | --- |
| 2e-06 | -9 |
| 3e-06 | -8.997 |
| 5e-06 | -8.994 |
| 9e-06 | -8.991 |
| 1.7e-05 | -8.988 |
| 2.9e-05 | -8.98499 |
| 4.9e-05 | -8.98199 |
| 8.1e-05 | -8.97899 |
| 0.000132 | -8.97599 |
| 0.000212 | -8.97299 |
| 0.000333 | -8.96999 |
| 0.000512 | -8.96699 |
| 0.000773 | -8.96399 |
| 0.001144 | -8.96099 |
| 0.001662 | -8.95799 |
| 0.002367 | -8.95498 |
| 0.003307 | -8.95198 |
| 0.004532 | -8.94898 |
| 0.006092 | -8.94598 |
| 0.008032 | -8.94298 |
| 0.010387 | -8.93998 |
| 0.013175 | -8.93698 |
| 0.01639 | -8.93398 |
| 0.02 | -8.93098 |
| 0.023936 | -8.92798 |
| 0.028098 | -8.92497 |
| 0.032351 | -8.92197 |
| 0.036534 | -8.91897 |
| 0.040465 | -8.91597 |
| 0.04396 | -8.91297 |
| 0.046841 | -8.90997 |
| 0.048954 | -8.90697 |
| 0.050184 | -8.90397 |
| 0.050463 | -8.90097 |
| 0.04978 | -8.89797 |
| 0.048183 | -8.89496 |
| 0.045774 | -8.89196 |
| 0.042703 | -8.88896 |
| 0.039156 | -8.88596 |
| 0.035343 | -8.88296 |
| 0.031483 | -8.87996 |
| 0.027788 | -8.87696 |
| 0.02446 | -8.87396 |
| 0.021676 | -8.87096 |
| 0.019584 | -8.86796 |
| 0.018303 | -8.86495 |
| 0.017919 | -8.86195 |
| 0.018488 | -8.85895 |
| 0.02003 | -8.85595 |
| 0.022537 | -8.85295 |
| 0.025962 | -8.84995 |
| 0.030229 | -8.84695 |
| 0.035226 | -8.84395 |
| 0.040806 | -8.84095 |
| 0.046793 | -8.83795 |
| 0.052985 | -8.83494 |
| 0.059162 | -8.83194 |
| 0.065094 | -8.82894 |
| 0.070554 | -8.82594 |
| 0.075327 | -8.82294 |
| 0.079224 | -8.81994 |
| 0.082094 | -8.81694 |
| 0.083827 | -8.81394 |
| 0.084366 | -8.81094 |
| 0.083714 | -8.80794 |
| 0.081929 | -8.80493 |
| 0.079134 | -8.80193 |
| 0.075508 | -8.79893 |
| 0.07129 | -8.79593 |
| 0.066768 | -8.79293 |
| 0.062282 | -8.78993 |
| 0.05821 | -8.78693 |
| 0.054964 | -8.78393 |
| 0.052975 | -8.78093 |
| 0.052685 | -8.77793 |
| 0.054528 | -8.77492 |
| 0.058908 | -8.77192 |
| 0.066182 | -8.76892 |
| 0.076631 | -8.76592 |
| 0.090435 | -8.76292 |
| 0.107647 | -8.75992 |
| 0.128179 | -8.75692 |
| 0.151783 | -8.75392 |
| 0.17805 | -8.75092 |
| 0.206423 | -8.74792 |
| 0.236219 | -8.74491 |
| 0.26666 | -8.74191 |
| 0.296918 | -8.73891 |
| 0.326162 | -8.73591 |
| 0.353605 | -8.73291 |
| 0.378542 | -8.72991 |
| 0.400387 | -8.72691 |
| 0.418692 | -8.72391 |
| 0.433154 | -8.72091 |
| 0.443618 | -8.71791 |
| 0.45006 | -8.7149 |
| 0.452574 | -8.7119 |
| 0.451349 | -8.7089 |
| 0.446641 | -8.7059 |
| 0.438755 | -8.7029 |
| 0.428018 | -8.6999 |
| 0.414763 | -8.6969 |
| 0.399307 | -8.6939 |
| 0.381948 | -8.6909 |
| 0.362955 | -8.6879 |
| 0.342573 | -8.68489 |
| 0.32103 | -8.68189 |
| 0.298554 | -8.67889 |
| 0.275387 | -8.67589 |
| 0.251798 | -8.67289 |
| 0.228101 | -8.66989 |
| 0.204652 | -8.66689 |
| 0.18185 | -8.66389 |
| 0.160126 | -8.66089 |
| 0.139924 | -8.65789 |
| 0.12168 | -8.65488 |
| 0.105798 | -8.65188 |
| 0.092626 | -8.64888 |
| 0.082442 | -8.64588 |
| 0.075434 | -8.64288 |
| 0.071694 | -8.63988 |
| 0.071216 | -8.63688 |
| 0.073888 | -8.63388 |
| 0.079501 | -8.63088 |
| 0.087749 | -8.62788 |
| 0.098243 | -8.62487 |
| 0.110517 | -8.62187 |
| 0.12405 | -8.61887 |
| 0.138286 | -8.61587 |
| 0.152661 | -8.61287 |
| 0.166634 | -8.60987 |
| 0.17972 | -8.60687 |
| 0.191525 | -8.60387 |
| 0.201766 | -8.60087 |
| 0.210295 | -8.59787 |
| 0.217098 | -8.59486 |
| 0.22229 | -8.59186 |
| 0.226088 | -8.58886 |
| 0.228779 | -8.58586 |
| 0.230676 | -8.58286 |
| 0.232078 | -8.57986 |
| 0.233231 | -8.57686 |
| 0.234308 | -8.57386 |
| 0.235396 | -8.57086 |
| 0.23651 | -8.56786 |
| 0.237615 | -8.56485 |
| 0.238664 | -8.56185 |
| 0.239631 | -8.55885 |
| 0.240548 | -8.55585 |
| 0.241522 | -8.55285 |
| 0.242735 | -8.54985 |
| 0.244423 | -8.54685 |
| 0.246839 | -8.54385 |
| 0.250202 | -8.54085 |
| 0.254641 | -8.53785 |
| 0.260148 | -8.53484 |
| 0.266547 | -8.53184 |
| 0.273482 | -8.52884 |
| 0.280441 | -8.52584 |
| 0.286793 | -8.52284 |
| 0.291858 | -8.51984 |
| 0.294981 | -8.51684 |
| 0.295607 | -8.51384 |
| 0.293353 | -8.51084 |
| 0.288053 | -8.50784 |
| 0.279786 | -8.50483 |
| 0.268864 | -8.50183 |
| 0.255807 | -8.49883 |
| 0.241286 | -8.49583 |
| 0.22605 | -8.49283 |
| 0.210855 | -8.48983 |
| 0.196388 | -8.48683 |
| 0.18321 | -8.48383 |
| 0.171704 | -8.48083 |
| 0.162061 | -8.47783 |
| 0.154278 | -8.47482 |
| 0.148181 | -8.47182 |
| 0.143461 | -8.46882 |
| 0.13973 | -8.46582 |
| 0.136577 | -8.46282 |
| 0.133615 | -8.45982 |
| 0.130529 | -8.45682 |
| 0.127105 | -8.45382 |
| 0.123241 | -8.45082 |
| 0.118947 | -8.44782 |
| 0.114318 | -8.44481 |
| 0.109514 | -8.44181 |
| 0.104718 | -8.43881 |
| 0.100108 | -8.43581 |
| 0.095824 | -8.43281 |
| 0.091952 | -8.42981 |
| 0.088516 | -8.42681 |
| 0.085482 | -8.42381 |
| 0.08277 | -8.42081 |
| 0.080273 | -8.41781 |
| 0.077876 | -8.4148 |
| 0.075479 | -8.4118 |
| 0.073005 | -8.4088 |
| 0.070415 | -8.4058 |
| 0.067706 | -8.4028 |

**Figure S76.** Total and partial density of states (oxo groups in sites A and B) in **{Mo_16_(Isop)_2_}**.

When considering the two-electron reductions it will be reasonable to assume that the first hydrogen will be added either to a Se, oxo group or molybdenum site. Starting with the **{Mo_8_Ox}** species the results of this exploration are summarised in Figure S78. The calculated reduction potentials show that by far the most favourable initial reduction and hydrogen addition will take place at the oxo site. This is consistent with the orbital make-up that was shown previously and there is also precedent for it in the literature.^[16]^ The added electron occupies a single Mo site as shown by a nearly unitary spin density. The metal pair adopts the well-defined oxidation states Mo(V)/Mo(IV) instead of a bond with added multiplicity. This is due to the asymmetry of the hydroxylation process.

Considering the second generation of proton/electron addition we reach potentials that are more similar for every site, but the most favourable isomer has the hydrogen atom bound to the selenium bridge (E^O^ = -0.82 V vs SHE). This doubly reduced species is now closed shell and does have some mixed valence character. The HOMO (Figure S78) does show some polarisation in the newly formed double bond towards the hydroxylated molybdenum.

The proximity of the two hydrogen moieties makes them ideal for a bimolecular (Tafel) mechanism of hydrogen evolution, and this will be further explored down below.

{Mo_8_Ox}H

{Mo_8_Ox}H_2_

**Figure S77.** Calculated proton coupled reduction potentials of **{Mo_8_Ox}**.

**Figure S78.** HOMO of the doubly reduced (2H^+^/2e^–^) **{Mo_8_Ox}** anion.

To contrast the aforementioned values with the sulfur analogue of {Mo_8_Ox} calculations of the half-pair reduction potentials were also done with this species. The results are summarised on Figure S79.

{Mo_8_S_8_Ox}H

{Mo_8_S_8_Ox}H_2_

**Figure S79.** Calculated proton coupled reduction potentials of the sulfur analogue of **{Mo_8_Ox}**.

Aside from the first protonation row, very little distinction can be made between the current and the previous values in Figure S77. The striking potential difference of -1.32 vs -3.60 V is consistent with the sulfur bridge displaying a higher proton affinity than selenium. Apart from that aspect there is nothing noticeably different between the two anions. Regarding the {Mo_16_(Isop)_2_} cluster, it is a reasonable assumption that the first tier of proton coupled electron transfer will take place at the oxo group given the previous results for **{Mo_8_Ox}**. Therefore, to render the computations expedient only three variations of the second H^+^/e^-^ addition were attempted. Two sites, one bearing a coordinatively saturated metal site (A) and the remainder (B) were used in the trials, and the results are summarised in Figure S81 and Figure S82. It is immediately evident that this cluster has a very different electrochemical behaviour relative to **{Mo_8_Ox}**. In site A two isomers nearly equal in energy (E^O^=-0.85V/-0.86V) are formed, these are designated 1 and 2. These are the species that will be considered in the mechanistic studies each having a separate pathway. The third species producing a terminal aqua ligand will be a dead end and is also less easy to form thermodynamically.

**Figure S80.** Calculated proton coupled reduction potentials of **{Mo_16_(Isop)_2_}** site A (circled in black).

**Figure S81.** Calculated proton coupled reduction potentials of **{Mo_16_(Isop)_2_}** site B (circled in black).

Regarding the B sites it is apparent that the electrochemical potentials required for the uptake of hydrogen are more negative than those of sites A by 12 mV. This is entirely consistent with the partial density of states shown in Figure S76 where the oxo moieties of sites A are more nucleophilic than those in sites B. Of the nearly degenerate isomers (structures 3 and 4) only one will be chemically viable for hydrogen generation. The aqua ligand is a chemical dead end.

The mechanism for HER was sought from the most stable reduced structures. A unimolecular (Volmer) type mechanism was sought but even after several constraints were placed on a proximal hydronium (H_3_O^+^) species we did not encounter any transition state or the desired products due to the extreme basicity of the selenium moieties which rapidly bind any neighbouring protons. The hydronium either releases one proton to the selenium bridge or to the adjacent oxo group of the opposite molybdenum. Having exhausted these possibilities we turned our attention to the bimolecular (Tafel) mechanistic route given that many of the previously screened isomers bear ideally positioned adjacent hydrogen atoms.

The **{Mo_8_Ox}H_2_** resting state should be the isomer generated with a half-pair potential E^O^=-0.82V (Figure S77) as this is the most thermodynamically favourable structure. The thermal activation barrier height is 7.9 kcal mol^-1^ which is quite good for a catalytic process. The HER on **{Mo_16_(Isop)_2_}** should be considered with isomers 1 and 2 which are equally viable.

**Figure S82.** Transition states leading to the formation of molecular hydrogen catalysed by **{Mo_8_Ox}** (top) and **{Mo_16_(Isop)_2_}** (bottom) accompanied by their respective HOMOs. Essential bond lengths (in Å) are listed in bold print.

Based on the above data we can conclude that the HER process in the case of **{Mo_16_(Isop)_2_}** is hampered due to the existence of coordination vacancies which raise the thermodynamic requirements for the electrochemical generation of the reduced active species. Furthermore, there is a mechanistic divergence due to the generation of side products (di-hydroxyl units) which inhibit the HER. Chemoselectivity of the di-protonated and di-reduced ring clusters is an essential requisite for successful HER catalysis. Selenium does play a significant part in this by acquiring highly basic features allowing it to act as a hydrogen acceptor. The matter becomes more complicated when there is competition with the oxo groups which show a slower activation pathway.

**11. References**

1. G. M. Sheldrick, *Acta Crystallogr. C Struct. Chem.* **2015**, 71, 3–8.
2. L. J. Farrugia, *J. Appl. Crystallogr*. **1999**, *32*, 837–838.
3. R. C. Clark, J. S. Reid, *Acta Crystallogr. A* **1995**, *51*, 887–897.
4. E. Cadot, B. Salignac, J. Marrot, A. Dolbecq, F. Sécheresse, Chem. Commun. **2000**, 10, 261–262.
5. E. Cadot, B. Salignac, S. Halut, F. Sécheresse, Angew. Chem. Int. Ed. **1998**, 37, 611–613.
6. H A. Dolbecq, E. Cadot and F. Sécheresse, Chem. Commun. **1998**, *21*, 2293–2294.
7. B. Salignac, S. Riedel, A. Dolbecq, F. Sécheresse, E. Cadot, J. Am. Chem. Soc. **2000**, 122, 10381–10389.
8. M.-M. Rohmer, L. Nadjo, J. Phys. Chem. C **2008**, 112, 1109–1114.
9. J.-F. Lemonnier, S. Floquet, A. Kachmar, M.-M. Rohmer, M. Bénard, J. Marrot, E. Terazzi, C. Piguet, E. Cadot, Dalton Trans. 2007, *28*, 3043–3054.
10. J.-F. Lemonnier, A. Kachmar, S. Floquet, J. Marrot, M.-M. Rohmer, M. Bénard, E. Cadot, Dalton Trans. 2008, *34*, 4565–4569.
11. H. Zang, H. N. Miras, J. Yan, D. L. Long, L. Cronin, J. Am. Chem. Soc. 2012, *134*, 11376–11379.
12. J.-F. Lemonnier, S. Floquet, J. Marrot, E. Terazzi, C. Piguet, P. Lesot, A. Pinto, E. Cadot, Chem. Eur. J. 2007, *13*, 3548–3557.
13. J. Marrot, C. Falaise, M. A. Fadel, N. Leclerc, M. Haouas, S. Floquet, F. Sécheresse, E. Cadot, Inorg. Chem. 2025, *64*, 4017–4024.
14. Wiley Science Solutions, https://sciencesolutions.wiley.com
15. A. Elliott, J. McAllister, L. Masaityte, M. Segado-Centellas, D. L. Long, A. Y. Ganin, Y.-F. Song, C. Bo, H. N. Miras, Chem. Commun. 2022, *58*, 6906.
16. C. Falaise, G. Mpacko Priso, N. Leclerc, M. Haouas, E. Cadot, *Inorg. Chem.* **2023**, *62*, 2494-2502.
17. F. Neese,'Software Update: The ORCA Program System - Version 6.0', *WIREs Comp. Molec. Sci.* **2025**, *15*, e70019.
18. F. Neese,'Software update: The ORCA program system—Version 5.0', *WIREs Comput. Mol. Sci.* **2022**, *12*, e1606.
19. F. Neese, F. Wennmohs, U. Becker, C. Riplinger, *J. Chem. Phys.* **2020**, *152*, 224108.
20. V. N. Staroverov, G. E. Scuseria, J. Tao, J. P. Perdew, *J. Chem. Phys.* **2003**, *119*, 12129-12137.
21. E. Caldeweyher, S. Ehlert, A. Hansen, H. Neugebauer, S. Spicher, C. Bannwarth, S. Grimme, *J. Chem. Phys.* **2019**, *150*.
22. A. Schafer, H. Horn, R. Ahlrichs, *J. Chem. Phys.* **1992**, *97*, 2571-2577.
23. A. Schafer, C. Huber, R. Ahlrichs, *J. Chem. Phys.* **1994**, *100*, 5829-5835.
24. D. Andrae, U. Häussermann, M. Dolg, H. Stoll, H. Preuss, *Theoret. Chem. Acc.* **1990**, *77*, 123-141.
25. F. Neese, *J. Comput. Chem.* **2003**, *24*, 1740-1747.
26. F. Neese, F. Wennmohs, A. Hansen, U. Becker, *Chem. Phys.* **2009**, *356*, 98-109.
27. S. Kossmann, F. Neese, *Chem. Phys. Lett.* **2009**, *481*, 240-243.
28. R. Izsák, F. Neese, *J. Chem. Phys.* **2011**, *135*, 144105.
29. B. Helmich-Paris, B. Souza, F. Neese, R. Izsák, *J. Chem. Phys.* **2021**, *155*, 104109.
30. F. Neese, *J. Comput. Chem.* **2023**, *44*, 381-396.
31. F. Weigend, *Phys. Chem. Chem. Phys.* **2006**, *8*, 1057-1065.
32. V. Barone, M. Cossi, *J. Phys. Chem. A* **1998**, *102*, 1995-2001.
33. M. Garcia-Ratés, F. Neese, *J. Comput. Chem.* **2019**, *40*, 1816-1828.
34. M. Garcia-Ratés, F. Neese, *J. Comput. Chem.* **2020**, *41*, 922-939.
35. C. P. Kelly, C. J. Cramer, D. G. Truhlar, *J. Phys. Chem. B* **2007**, *111*, 408-422.
36. M. D. Tissandier, K. A. Cowen, W. Y. Feng, E. Gundlach, M. H. Cohen, A. D. Earhart, J. V. Coe, T. R. Tuttle, *J. Phys. Chem. A* **1998**, *102*, 7787-7794.
37. C. P. Kelly, C. J. Cramer, D. G. Truhlar, *J. Phys. Chem. B* **2006**, *110*, 16066-16081.
